# Prediction of early-stage melanoma recurrence using clinical and histopathologic features

**DOI:** 10.1038/s41698-022-00321-4

**Published:** 2022-10-31

**Authors:** Guihong Wan, Nga Nguyen, Feng Liu, Mia S. DeSimone, Bonnie W. Leung, Ahmad Rajeh, Michael R. Collier, Min Seok Choi, Munachimso Amadife, Kimberly Tang, Shijia Zhang, Jordan S. Phillipps, Ruple Jairath, Nora A. Alexander, Yining Hua, Meng Jiao, Wenxin Chen, Diane Ho, Stacey Duey, István Balázs Németh, Gyorgy Marko-Varga, Jeovanis Gil Valdés, David Liu, Genevieve M. Boland, Alexander Gusev, Peter K. Sorger, Kun-Hsing Yu, Yevgeniy R. Semenov

**Affiliations:** 1 Department of Dermatology, Massachusetts General Hospital, Harvard Medical School, Boston, MA USA; 2 Department of Biomedical Informatics, Harvard Medical School, Boston, MA USA; 3 Department of Systems Biology, Harvard Medical School, Boston, MA USA; 4 School of Systems and Enterprises, Stevens Institute of Technology, Hoboken, NJ USA; 5 Department of Pathology, Brigham and Women’s Hospital, Harvard Medical School, Boston, MA USA; 6 Department of Dermatology and Allergology, University of Szeged, Szeged, Hungary; 7 Department of Translational Medicine, Lund University, Lund, Sweden; 8 Department of Medicine, Dana-Farber Cancer Institute, Boston, MA USA

**Keywords:** Melanoma, Translational research, Risk factors

## Abstract

Prognostic analysis for early-stage (stage I/II) melanomas is of paramount importance for customized surveillance and treatment plans. Since immune checkpoint inhibitors have recently been approved for stage IIB and IIC melanomas, prognostic tools to identify patients at high risk of recurrence have become even more critical. This study aims to assess the effectiveness of machine-learning algorithms in predicting melanoma recurrence using clinical and histopathologic features from Electronic Health Records (EHRs). We collected 1720 early-stage melanomas: 1172 from the Mass General Brigham healthcare system (MGB) and 548 from the Dana-Farber Cancer Institute (DFCI). We extracted 36 clinicopathologic features and used them to predict the recurrence risk with supervised machine-learning algorithms. Models were evaluated internally and externally: (1) five-fold cross-validation of the MGB cohort; (2) the MGB cohort for training and the DFCI cohort for testing independently. In the internal and external validations, respectively, we achieved a recurrence classification performance of AUC: 0.845 and 0.812, and a time-to-event prediction performance of time-dependent AUC: 0.853 and 0.820. Breslow tumor thickness and mitotic rate were identified as the most predictive features. Our results suggest that machine-learning algorithms can extract predictive signals from clinicopathologic features for early-stage melanoma recurrence prediction, which will enable the identification of patients that may benefit from adjuvant immunotherapy.

## Introduction

Despite recent therapeutic advances in the treatment of advanced-stage melanomas, the number of melanoma deaths in the United States is estimated to exceed 90,000 over the next decade^1^. Emerging evidence suggests that the majority of melanoma mortality occurs in patients with a recurrence of the disease that was early-stage (Stage I or Stage II) at the time of diagnosis^2^. However, the recurrence of melanoma is typically not detected until the onset of symptomatic metastatic progression. Thus, there is a critical need for accurate prognostic tools to identify high-risk patients who would benefit from enhanced surveillance^3^. Additionally, identifying high-risk patients can support clinicians in determining who should receive adjuvant immunotherapy, which was recently approved for early-stage melanomas^4^. This decision is particularly important as immunotherapy has been associated with a high rate of morbid and potentially fatal immune-related adverse events (irAEs) occurring in up to 40% of treated patients^5–7^. Accordingly, balancing potential benefits with these risks requires a reliable understanding of which patients are most likely to experience disease recurrence and thereby would be most likely to benefit from early systemic therapy. Overall, early detection of melanoma recurrence can help minimize the number of patients exposed to treatment toxicities, prevent metastatic progression, and improve patient survival.

Research to date on early-stage melanoma recurrence has been limited by the sample size of individual studies^8,9^, duration of follow-up^10,11^, or access to well-phenotyped cohorts with detailed patient and tumor characteristics^11,12^. As a result, it has been difficult to reliably determine disease recurrence status and assess downstream outcomes in this population. In the present study, we leverage detailed multi-institutional registries across the Mass General Brigham healthcare system (MGB) and the Dana-Farber Cancer Institute (DFCI) to develop risk prediction models for early-stage melanoma recurrence. As the largest providers of dermatology services in the state of Massachusetts, these hospital systems offer access to detailed clinical features and tumor characteristics from a large population of patients who were diagnosed with melanoma both within and outside of the academic setting.

We build on prior literature on this topic by incorporating a wide array of clinical and histopathologic variables in our risk prediction assessment^10,12–19^. With the intent to stratify melanoma by survival outcomes, the American Joint Committee on Cancer (AJCC) melanoma staging system was established based on analyses of a large international melanoma database, which determined that Breslow thickness, ulceration, and historically mitotic rate were the most important prognostic factors in patients with localized melanoma^16,17^. Patient age at diagnosis and anatomic site of the primary melanoma were also shown to be significantly associated with recurrence of primary melanoma in separate investigations^15,18,20^. Furthermore, a recent study suggested that the presence of lymphovascular invasion and the baseline autoimmune disease were also independently associated with melanoma recurrence^10^. However, there is insufficient information on the cumulative predictive capacity of these features in prognosticating melanoma recurrence. To address this knowledge gap, we propose a comprehensive risk stratification approach using machine-learning modeling of a wide range of clinical and tumor synoptic features from EHRs. Similar approaches have been previously applied with success in other cancer settings^21^, achieving the area under the receiver operating characteristic curve (AUC) of 0.79–0.80^22,23^. To the best of our knowledge, our study presents a comprehensive analysis of melanoma recurrence combining state-of-the-art machine-learning techniques and granular EHR data. The primary goal of this study is to build machine-learning models for reliable prediction of early-stage melanoma recurrence and identification of significant independent risk factors using the extracted clinical and histopathologic features. Specifically, we performed two types of prediction: (1) recurrence vs. non-recurrence classification; (2) time-to-event recurrence risk prediction.

## Results

### Patient characteristics

**Fig. 1:**
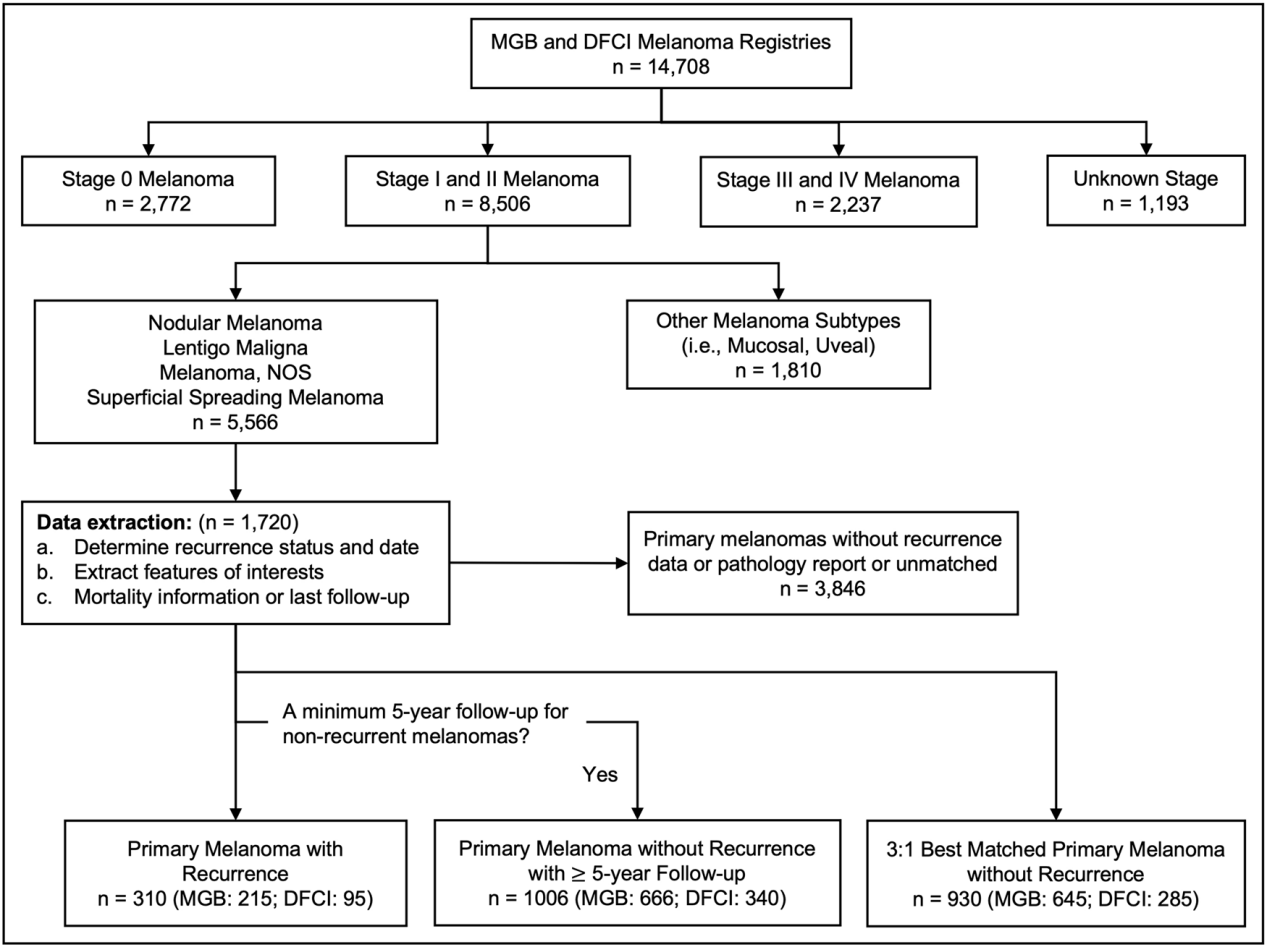
MGB and DFCI primary melanoma registry design and cohort definitions. Non-recurrent melanomas were categorized into two groups: one group with a minimum of 5-year follow-up duration; another group 3:1 best matched to the recurrent melanomas in terms of follow-up duration. The first group was compared to recurrent melanomas in the binary recurrence classification tasks. The second group was compared to recurrent melanomas in the time-toevent recurrence prediction tasks.

We identified 1720 stage I/II cutaneous melanomas, with 1172 (68%) melanomas from MGB and 548 (32%) melanomas from DFCI (Fig. 1), that were diagnosed between 2000 and 2020. The non-recurrent melanomas were categorized into two groups: one group with a minimum of 5-year follow-up duration (to minimize the risk of false non-recurrences); another group 3:1 best matched to the recurrent melanomas in terms of follow-up duration. The first group was compared to recurrent melanomas for the binary recurrence classification tasks. The second group was compared to recurrent melanomas for the time-to-event recurrence prediction tasks. Comprehensive clinical and histopathologic features were extracted from EHRs (Supplementary Table 1).

**Fig. 2:**
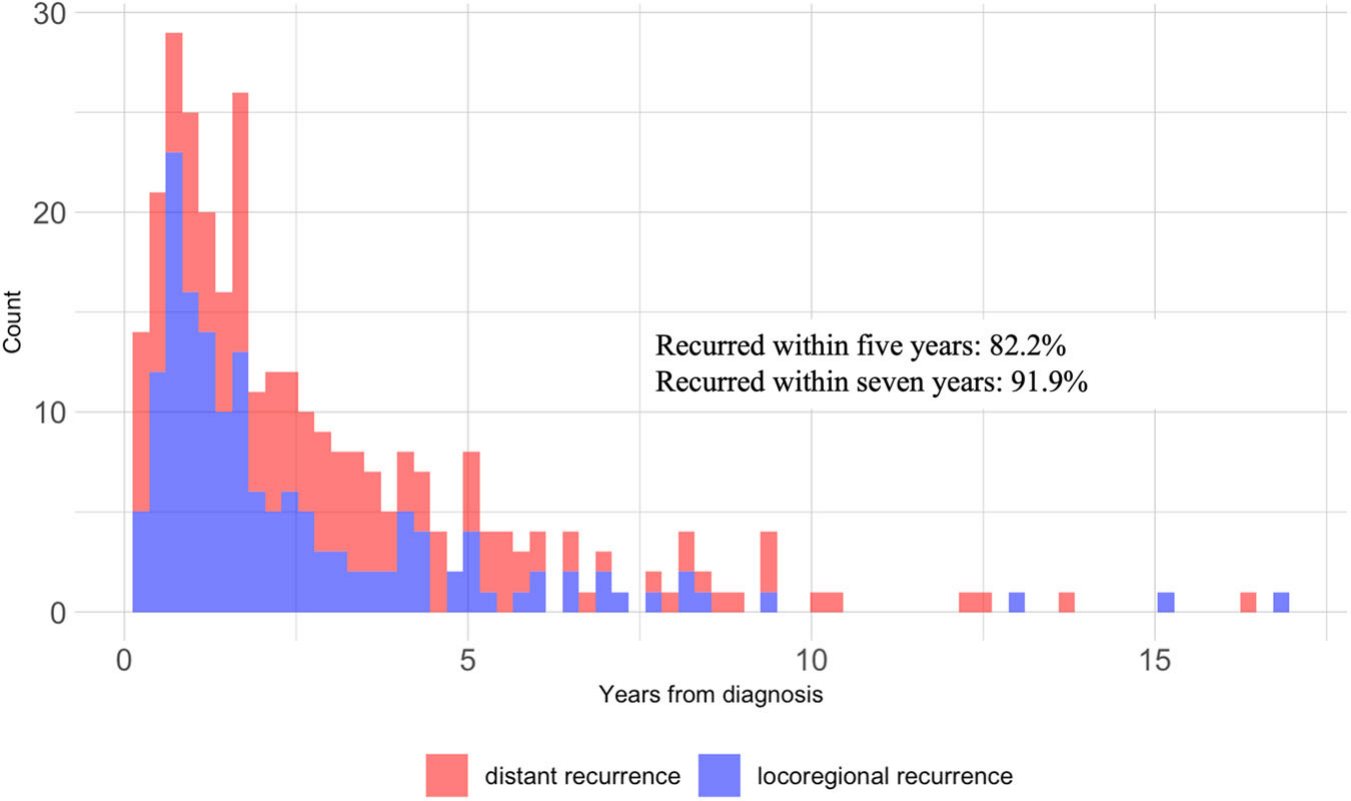
Distribution of recurrent melanomas in the study population. Among the 310 recurrent melanomas, 255 (82%) recurred within five years and 285 (92%) recurred within 7 years of primary melanoma diagnosis.

The basic patient characteristics of the entire study population are described in Table 1. All characteristics are detailed in Supplementary Table 3. The median follow-up for the entire cohort was 7.2 (IQR: 3.6–11.6) years. Overall, 310 out of 1720 (18%) melanomas recurred, among which 151 (48.7%) were distant recurrences. The median time from diagnosis to recurrence was 1.9 (IQR: 0.9–3.9) years. There was a small difference in the distribution of the year of diagnosis between the recurrent group and the non-recurrent group (p-value: 0.02). However, the mean and median years of diagnosis were identical for both groups (2010). The recurrent group had a higher mortality rate (49% vs. 22%, *p*-value <0.001) and was older at the time of diagnosis (65 vs. 60 years old, *p*-value <0.001) when compared to the non-recurrent group. The percentage of males in the recurrent group was higher (64% vs. 55%, *p*-value: 0.004) than in the non-recurrent group. Most of the population self-identified as Caucasian in both groups (recurrence: 98%; non-recurrence: 99%). Among the 310 recurrent melanomas, the respective number of cases that recurred within 5 years and 7 years after the diagnosis of the primary melanoma is 255 (82%) and 285 (92%) (Fig. 2). The majority of the entire cohort (74% for recurrences and 95% for non-recurrences) was with negative or not indicated regional lymph node histology. There were more patients in the recurrence group who didn’t have the sentinel lymph node biopsy performed due to age or comorbidity (19%) than in the non-recurrence group (2%).

**Table 1:** Characteristics of the study population.

Characteristic	Non-recurrence (*N* = 1410)	Recurrence (*N* = 310)	*p*-value
Institution			
DFCI	453 (32.1%)	95 (30.6%)	0.660
MGB	957 (67.9%)	215 (69.4%)	
Duration of follow-up (year)			
Median [IQR]	7.3 [3.8, 12.2]	5.8 [3.2, 9.3]	<0.001
Recurrence type			
Distant	Not applicable	151 (48.7%)	
Locoregional	Not applicable	159 (51.3%)	
Time to recurrence (year)			
Median [IQR]	Not applicable	1.9 [0.9, 3.9]	
Time to locoregional recurrence (year)			
Median [IQR]	Not applicable	1.5 [0.9, 3.1]	
Time to distant recurrence (year)			
Median [IQR]	Not applicable	2.5 [1.3, 4.5]	
Mortality status			
Alive	1106 (78.4%)	158 (51.0%)	<0.001
Dead	304 (21.6%)	152 (49.0%)	
Year of diagnosis			
2000–2005	364 (25.8%)	59 (19.0%)	0.020
2006–2010	388 (27.5%)	88 (28.4%)	
2011–2015	514 (36.5%)	117 (37.7%)	
2016–2020	144 (10.2%)	46 (14.8%)	
Age at diagnosis (year)			
Median [IQR]	60 [47, 70]	65 [55, 76]	<0.001
Sex			
Female	638 (45.2%)	112 (36.1%)	0.004
Male	772 (54.8%)	198 (63.9%)	
Race			
White	1399 (99.2%)	304 (98.1%)	0.122
Unavailable/Other	11 (0.8%)	6 (1.9%)	
Histology type			
Lentigo maligna melanoma	107 (7.6%)	23 (7.4%)	<0.001
Melanoma, NOS	269 (19.1%)	67 (21.6%)	
Nodular melanoma	115 (8.2%)	85 (27.4%)	
Superficial spreading melanoma	919 (65.2%)	135 (43.5%)	
Site			
Skin of face	147 (10.4%)	51 (16.5%)	<0.001
Skin of lower limb and hip	268 (19.0%)	65 (21.0%)	
Skin of scalp and neck	84 (6.0%)	46 (14.8%)	
Skin of trunk	542 (38.4%)	89 (28.7%)	
Skin of upper limb and shoulder	369 (26.2%)	59 (19.0%)	
AJCC Stage			
1A	862 (61.1%)	49 (15.8%)	<0.001
1B	386 (27.4%)	84 (27.1%)	
2A	71 (5.0%)	58 (18.7%)	
2B	62 (4.4%)	63 (20.3%)	
2C	29 (2.1%)	56 (18.1%)	
Breslow thickness			
Mean (SD)	1.0 (1.4)	3.0 (4.2)	<0.001
Median [IQR]	0.6 [0.04, 1.1]	2.0 [1.1, 3.9]	
Anatomic level			
Mean (SD)	3.2 (0.9)	4.0 (0.8)	<0.001
Median [IQR]	3.0 [2.0, 4.0]	4.0 [4.00, 4.0]	
Ulceration			
Absent	1305 (92.6%)	210 (67.7%)	<0.001
Present	101 (7.2%)	99 (31.9%)	
Unavailable	4 (0.3%)	1 (0.3%)	
Mitotic rate (mitoses/mm^2^)			
Mean (SD)	1.8 (3.5)	7.6 (11.0)	<0.001
Median [IQR]	1.0 [0.0, 2.0]	4.0 [2.0, 10.0]	
Regional lymph node histology			
Not indicated	826 (58.6%)	55 (17.7%)	<0.001
All nodes negative	519 (36.8%)	175 (56.5%)	
Not performed: unknown reason	30 (2.1%)	12 (3.9%)	
Not performed: due to age/comorbidity	30 (2.1%)	59 (19.0%)	
Not performed: deferred by patient	5 (0.4%)	9 (2.9%)

The patient characteristics of the MGB and DFCI cohorts for the binary recurrence classification tasks are shown in Supplementary Table 4. In the MGB cohort, the median follow-up was 11.4 (IQR: 7.2–15.1) years, and 215 of 881 (24%) melanomas recurred, among which 98 (46%) cases were distant recurrences. The median time from diagnosis to recurrence was 1.9 (IQR: 0.9–4.2) years. In the DFCI cohort, the median follow-up was 7.0 (IQR: 6.1–8.1) years, and 95 of 435 (22%) melanomas recurred, among which 53 (56%) cases were distant recurrences. The median time from diagnosis to recurrence was 1.8 (IQR: 1.1–3.5) years.

The patient characteristics of the MGB and DFCI cohorts for time-to-event recurrence prediction tasks are shown in Supplementary Table 5. The respective median follow-up duration for the MGB and DFCI cohorts was 5.9 (IQR: 1.9–11.3) years and 5.8 (IQR: 3.4–7.8) years. After the non-recurrent melanomas were identified by 3:1 best matching to the recurrent melanomas in terms of follow-up duration, there were no significant differences in follow-up duration and year of diagnosis between the recurrences and non-recurrences in each cohort.

### Melanoma recurrence classification

**Fig. 3:**
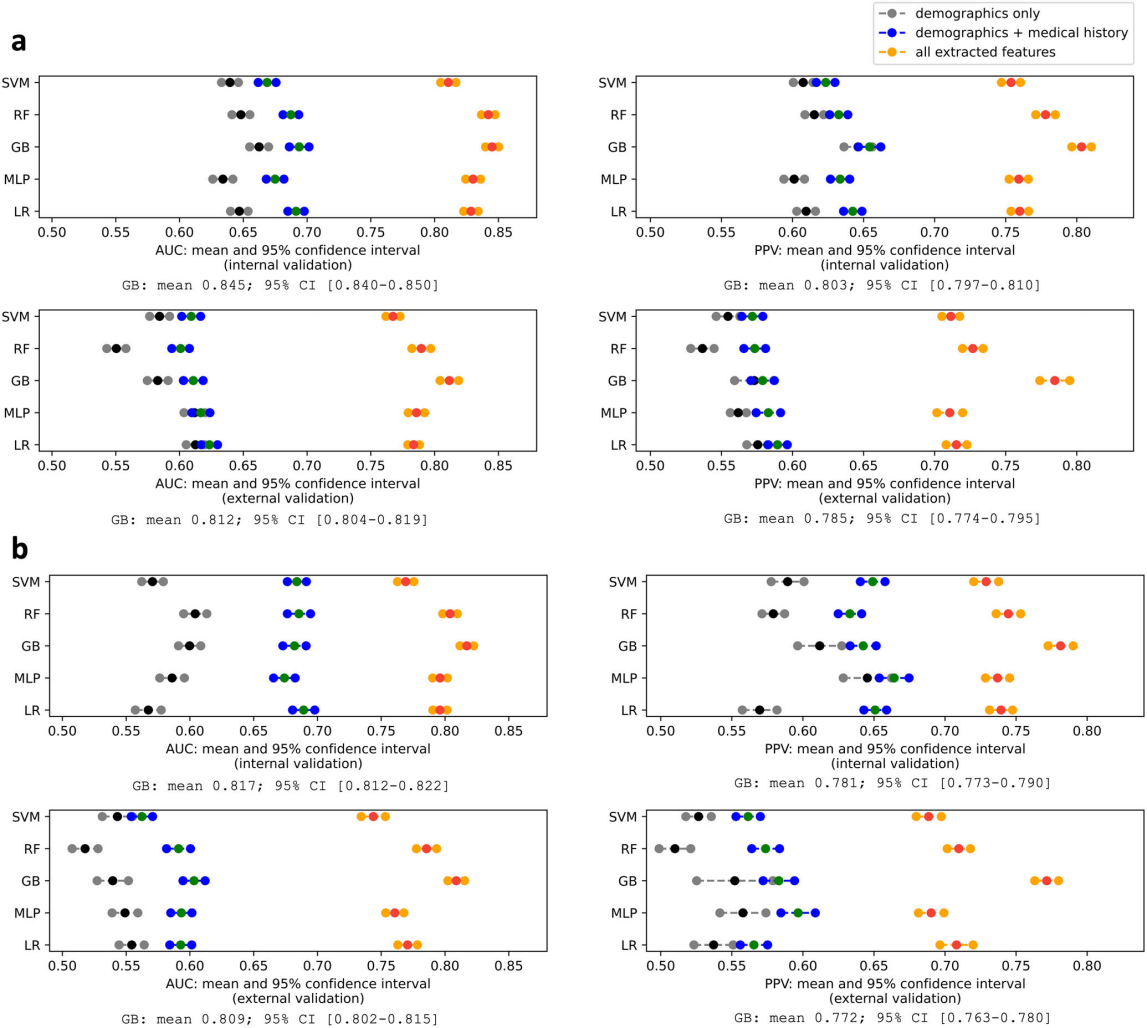
Model performances in the binary recurrence classification using patient demographics, medical history, and tumor characteristics. **a** AUC and PPV in internal validation (first row) and external validation (second row) when experimenting on the original cohorts (MGB: 215 recurrences vs. 666 non-recurrences; DFCI: 95 recurrences vs. 340 non-recurrences). **b** AUC and PPV in internal validation (first row) and external validation (second row) when experimenting on the core complete cohorts: negative or not indicated regional lymph node histology, known mitotic rate, and known ulceration (MGB: 142 recurrences vs. 410 non-recurrences; DFCI: 74 recurrences vs. 317 non-recurrences). The best model performance among SVM, RF, GB, MLP, and LR models is specified below each plot.

Melanomas were labeled as having a recurrence or not, and five machine-learning algorithms were applied to classify recurrent and non-recurrent melanomas. Recurrence prediction model performances with successive additions of patient demographics, medical history, and tumor characteristics are shown in Fig. 3a. The area under the receiver operating characteristic curve (AUC), positive predictive value (PPV), sensitivity, specificity, and accuracy of models that used all extracted features are detailed in Supplementary Table 6.

When using only demographic features, all models failed to yield an acceptable prediction performance (external AUC values <0.65). After adding medical history features, the AUC values were significantly improved in both internal and external validation for GB models (*p*-value: <0.01), while the PPV was not significantly improved (*p*-value: >0.05). After integrating all extracted tumor characteristics, both AUC and PPV were significantly improved for all models (*p*-value: <0.001), indicating that tumor characteristics were the most dominant features for the melanoma recurrence classification in our study.

When using all extracted features, GB models achieved the highest AUC in both internal (0.845; 95% CI: 0.840–0.850) and external (0.812; 95% CI: 0.804–0.819) validations. Additionally, GB models achieved the highest performance in terms of PPV (internal: 0.803; 95% CI: 0.797–0.810; external: 0.785; 95% CI: 0.774–0.795), and accuracy (internal: 0.772; 95% CI: 0.768–0.777; external: 0.741; 95% CI: 0.735–0.746) (Supplementary Table 6). The AUC and PPV of the GB model in the external validation are significantly different from other models (*p*-value < 0.001). SVM models did not achieve competitive performance in both internal and external validations.

In addition to experiments on the original MGB and DFCI cohorts (Supplementary Table 4), we also conducted sensitivity analyses using subgroups with no missing values for core tumor features (histologic type, tumor site, AJCC stage, Breslow thickness, anatomic level, laterality, mitotic rate, and ulceration), and only negative sentinel lymph nodes or not indicated sentinel lymph node biopsy (labeled “core complete cohorts”). There were 142 recurrences vs. 410 non-recurrences in the MGB cohort, and 74 recurrences vs. 317 non-recurrences in the DFCI cohort. The results are presented in Fig. 3b. Compared to the original cohorts, the GB model achieved consistent performance in the external validation (AUC: 0.812 vs. 0.809, *p*-value: 0.577, PPV: 0.785 vs. 0.772, *p*-value: 0.055).

**Fig. 4:**
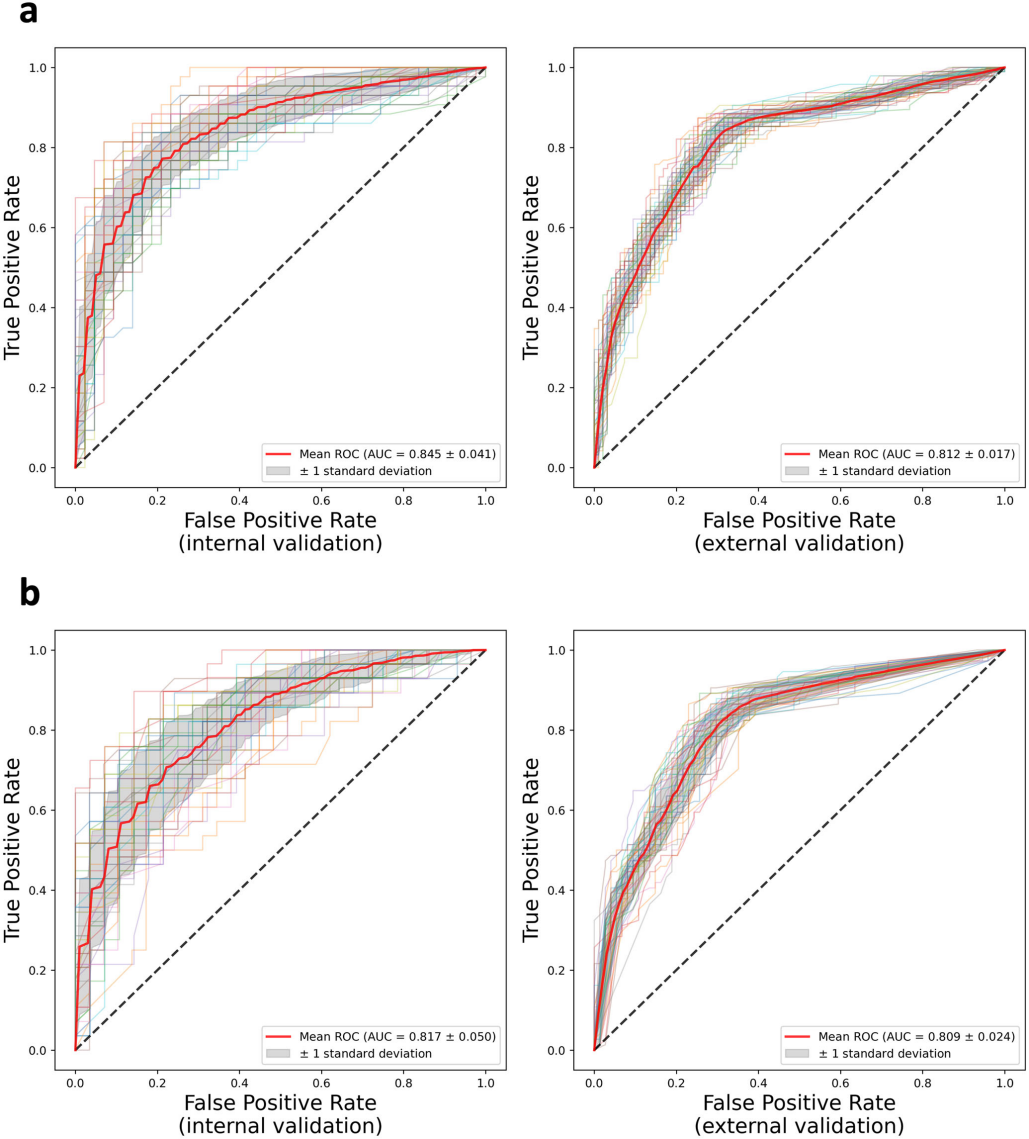
ROC curves with 50 repeats for the binary recurrence classification by GB models. **a** ROC curves in internal and external validations with the original cohorts. **b** ROC curves in internal and external validations with the core complete cohorts: negative or not indicated regional lymph node histology, known mitotic rate, and known ulceration. Consistent results between the original and core complete cohorts are achieved.

The receiver operating characteristic (ROC) curves using GB models are shown in Fig. 4. Each experiment was repeated 50 times. All 50 ROC curves, the mean ROC curve, and the standard deviation are presented. The curves in Fig. 4a, b demonstrated similar shapes. The ROC curves for RF, LR, MLP, and SVM are presented in Supplementary Fig. 2.

### Sensitivity analyses for recurrence classification

In the original MGB and DFCI cohorts (Supplementary Table 4), a 5-year minimum follow-up duration for non-recurrent melanomas was applied to reduce the likelihood of false non-recurrence. We further performed recurrence vs. non-recurrence classification experiments using GB and RF models when the minimum follow-up duration for non-recurrent melanomas was 7 years (labeled “seven years”) and when all tumor features and median income were without missing values, and regional lymph node histology was negative or not indicated (labeled “complete cohorts”). The sample sizes and results are summarized in Table 2.

**Table 2:** Sensitivity analyses for recurrence classification by GB and RF models.

Data	Sample size	Validation	GB		RF	
			AUC	PPV	AUC	PPV
Original cohorts (5 years)^a^	MGB: 215 recurrences 666 non-recurrences DFCI: 95 recurrences 340 non-recurrences	Internal	0.845 CI^f^:0.840–0.850	0.803 CI:0.797–0.810	0.842 CI:0.837–0.847	0.778 CI:0.771–0.785
		External	0.812 CI:0.804–0.819	0.785 CI:0.774–0.795	0.790 CI:0.782–0.797	0.737 CI:0.727–0.748
		*p*-value^e^	<0.001	0.021	<0.001	0.422
Core complete cohorts^b^	MGB: 142 recurrences 410 non-recurrences DFCI: 74 recurrences 317 non-recurrences	Internal	0.817 CI:0.812–0.82	0.781 CI:0.773–0.79	0.804 CI:0.798–0.81	0.745 CI:0.736–0.753
		External	0.809 CI:0.802–0.815	0.772 CI:0.763–0.78	0.786 CI:0.778–0.793	0.733 CI:0.722–0.743
		*p*-value^e^	0.188	0.336	0.465	<0.001
Complete cohorts^c^	MGB: 116 recurrences 299 non-recurrences DFCI: 45 recurrences 193 non-recurrences	Internal	0.805 CI:0.799–0.812	0.785 CI:0.773–0.796	0.777 CI:0.771–0.783	0.741 CI:0.732–0.75
		External	0.752 CI:0.742–0.763	0.737 CI:0.723–0.752	0.730 CI:0.721–0.740	0.697 CI:0.684–0.71
		*p*-value^e^	<0.001	<0.001	<0.001	<0.001
Seven years^d^	MGB: 215 recurrences 578 non-recurrences DFCI: 95 recurrences 182 non-recurrences	Internal	0.851 CI:0.837–0.864	0.809 CI:0.794–0.825	0.852 CI:0.840–0.864	0.792 CI:0.777–0.807
		External	0.827 CI:0.822–0.833	0.793 CI:0.786–0.801	0.816 CI:0.811–0.821	0.759 CI:0.751–0.766
		*p*-value^e^	0.002	0.077	<0.001	<0.001

^a^The original cohorts (Supplementary Table 4) in which there were no missing values for sex, insurance type, age at diagnosis, HPCM, histological type, tumor site, stage, thickness, anatomic level, laterality (Supplementary Table 1), and the minimum follow-up duration for the non-recurrent melanoma was 5 years.

^b^The “core complete” cohorts were based on the original cohorts with no missing values for additional core features: negative or not indicated regional lymph node histology, no missing values for ulceration, and no missing values for mitotic rate.

^c^The “complete” cohorts were based on the core complete cohorts with additional constraints: no missing values for median income and all tumor characteristics.

^d^The “seven years” cohorts were based on the original cohorts in which the minimum follow-up duration for the non-recurrent melanomas was 7 years.

^e^*p*-value: comparison between the internal validation and the external validation.

^f^CI: 95% confidence interval.

In the complete cohorts, the number of recurrences in the MGB cohort was 116 and the number of recurrences in the DFCI cohort was 45. The prediction performance (internal AUC: 0.805, PPV: 0.785; external AUC: 0.752, PPV: 0.737) deteriorated significantly (*p*-values: <0.001) compared to the performance of the original analysis (internal AUC: 0.845, PPV: 0.803; external AUC: 0.812, PPV: 0.785). When the minimum follow-up duration for non-recurrent melanomas was 7 years, the GB model achieved slightly better results in the external validation (AUC: 0.827, PPV: 0.793, *p*-value: 0.001 for AUC; *p*-value: 0.175 for PPV) compared to the performance of the original analysis (AUC: 0.812, PPV: 0.785).

### Feature importance in recurrence classification

**Fig. 5:**
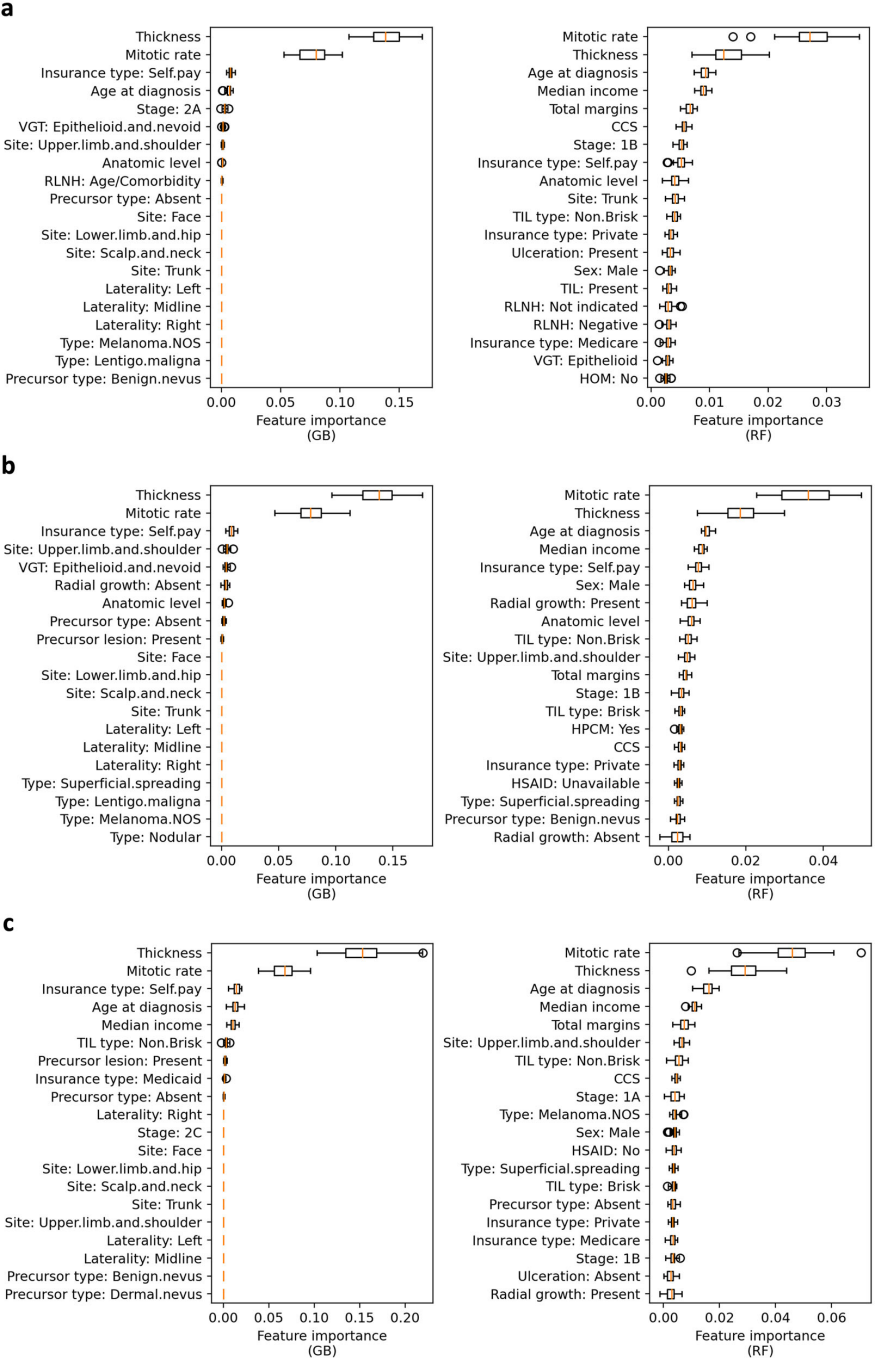
Feature importance in recurrence classification by RF and GB models. **a** The 20 most important features when experimenting on the original cohorts. **b** The 20 most important features when experimenting on the core complete cohorts: negative or not indicated regional lymph node histology, known mitotic rate, and known ulceration. **c** The 20 most important features when experimenting on the complete cohorts: negative or indicated regional lymph node histology, known median income, and all tumor features available. The box extends from the first quartile to the third quartile of the feature importance values for each feature, with a line at the median. The whiskers extend from the box by 1.5x the interquartile range. Flier points are those past the end of the whiskers.

We further examined the primary features used in the recurrence vs. non-recurrence classification by conducting permutation importance^24^ with each classifier. All features after one-hot encoding are described in Supplementary Table 2. The experiments were performed with 50 repeats and AUC used for scoring. Figure 5 and Supplementary Fig. 3 demonstrated the 20 most predictive features in the recurrence classification tasks with GB, RF (Fig. 5), MLP, LR, and SVM (Supplementary Fig. 3) models. Figure 5 showed that in the experiments on all three types of cohorts (original, core complete, and complete) by GB and RF models (best models), the two most important features were consistently Breslow thickness and mitotic rate. Other predictive features included insurance type, age at diagnosis, median income, AJCC stage, tumor site, total surgical margin, radial growth, and anatomic level. In the MLP, LR, and SVM models (Supplementary Fig. 3), the most important features included insurance type, mitotic rate, AJCC stage, anatomic level, tumor site, and sex. In these models, Breslow thickness was not among the top 10 important features.

Given the collinearity of anatomic level, Breslow thickness, AJCC stage, and ulceration, we also conducted the following experiments by (1) keeping AJCC stage (removing anatomic level, Breslow thickness, and ulceration) to examine the importance of AJCC stage; (2) by keeping Breslow thickness and ulceration (removing anatomic level and AJCC stage) to see the importance of ulceration. In these two experiments, all other unmentioned features were kept in the models. Results are presented in Supplementary Table 7 and Supplementary Fig. 4. Supplementary Table 7 showed that overall consistent performances were achieved. Supplementary Fig. 4 demonstrated that when anatomic level, Breslow thickness, and ulceration were removed, AJCC stage became one of the top two features in the GB model and became more important in the RF model. When anatomic level and AJCC stage were removed, ulceration became more important in the GB model compared to the results when all extracted features were used. In these experiments, mitotic rate remained as one of the two most important features.

### Time-to-event melanoma recurrence risk prediction

We built time-to-event models for melanoma recurrence risk prediction using four well-known machine-learning algorithms. Along with the original cohorts, we also conducted experiments on the “core complete” and the “complete” cohorts. The sample sizes and results are presented in Table 3 (GB-T and RF-T) and Supplementary Table 8 (Coxnet and CoxPH).

**Table 3:** Time-to-event melanoma recurrence prediction by GB-T and RF-T models.

Data	Sample size	Validation	GB-T		RF-T	
			Time-dependent AUC	Concordance index	Time-dependent AUC	Concordance index
Original cohorts^a^	MGB: 215 recurrences 645 non-recurrences DFCI: 95 recurrences 285 non-recurrences	Internal	0.853 CI^e^:0.849–0.857	0.820 CI:0.816–0.824	0.845 CI:0.841–0.850	0.813 CI:0.809–0.816
		External	0.820 CI:0.819–0.821	0.809 CI:0.808–0.810	0.810 CI:0.809–0.811	0.807 CI:0.807–0.808
		*p*-value^d^	<0.001	0.012	<0.001	0.248
Core complete cohorts^b^	MGB: 142 recurrences 516 non-recurrences DFCI: 74 recurrences 276 non-recurrences	Internal	0.829 CI:0.819–0.840	0.788 CI:0.783–0.793	0.788 CI:0.783–0.793	0.783 CI:0.775–0.791
		External	0.804 CI:0.802–0.806	0.808 CI:0.807–0.810	0.781 CI:0.778–0.783	0.793 CI:0.791–0.795
		*p*-value^d^	0.001	0.001	0.057	0.312
Complete cohorts^c^	MGB: 116 recurrences 365 non-recurrences DFCI: 45 recurrences 175 non-recurrences	Internal	0.808 CI:0.800–0.816	0.768 CI:0.760–0.777	0.795 CI:0.786–0.804	0.769 CI:0.761–0.776
		External	0.673 CI:0.671–0.675	0.752 CI:0.75–0.753	0.675 CI:0.67–0.68	0.734 CI:0.732–0.736
		*p*-value^d^	<0.001	0.007	<0.001	<0.001

^a^The original cohorts (Supplementary Table 5) in which there were no missing values for sex, insurance type, age at diagnosis, HPCM, histological type, tumor site, AJCC stage, Breslow thickness, anatomic level, and laterality (Supplementary Table 1).

^b^The “core complete” cohorts were based on the original cohorts with no missing values for additional core features: negative or not indicated regional lymph node histology, no missing value for ulceration, and no missing value for mitotic rate.

^c^The “complete” cohorts were based on the core complete cohorts with additional constraints: no missing values for median income and all tumor characteristics. Considering the small sample size for time-to-vent prediction, twofold cross-validation was used for internal validation.

^d^*p*-value: comparison between the internal validation and the external validation.

^e^CI: 95% confidence interval.

For the original cohorts, GB-T and RF-T models achieved better performance than Coxnet and CoxPH (*p*-value: <0.001). In both internal and external validations, the GB-T model achieved better time-dependent AUC (internal 0.853 vs. 0.845, *p*-value: 0.013; external 0.820 vs. 0.810, *p*-value: <0.001) and concordance index (internal 0.820 vs. 0.813, *p*-value: 0.007; external: 0.809 vs. 0.807, *p*-value: 0.045) than the RF-T model.

When experimenting on the core complete cohorts, the prediction performance of the GB-T model deteriorated significantly in the internal validation (time-dependent AUC: 0.853 vs. 0.829; concordance index: 0.820 vs. 0.788; *p*-values: <0.001) and decreased slightly in the external validation (time-dependent AUC: 0.820 vs. 0.804, *p*-value: <0.001; concordance index: 0.820 vs. 0.808; *p*-value: 686) compared to the performance of the original cohorts. In the complete cohorts, the number of recurrences was 116 in the MGB cohort and 45 in the DFCI cohort. The time-dependent AUC and concordance index of the GB-T model were further reduced in both internal (time-dependent AUC: 0.808, concordance index: 0.768) and external (time-dependent AUC: 0.673, concordance index: 0.752) validations. In the external validation, the best time-dependent AUC (0.692) achieved by the Coxnet and CoxPH models was significantly worse than the ones on the original cohorts (*p*-value: <0.001).

**Fig. 6:**
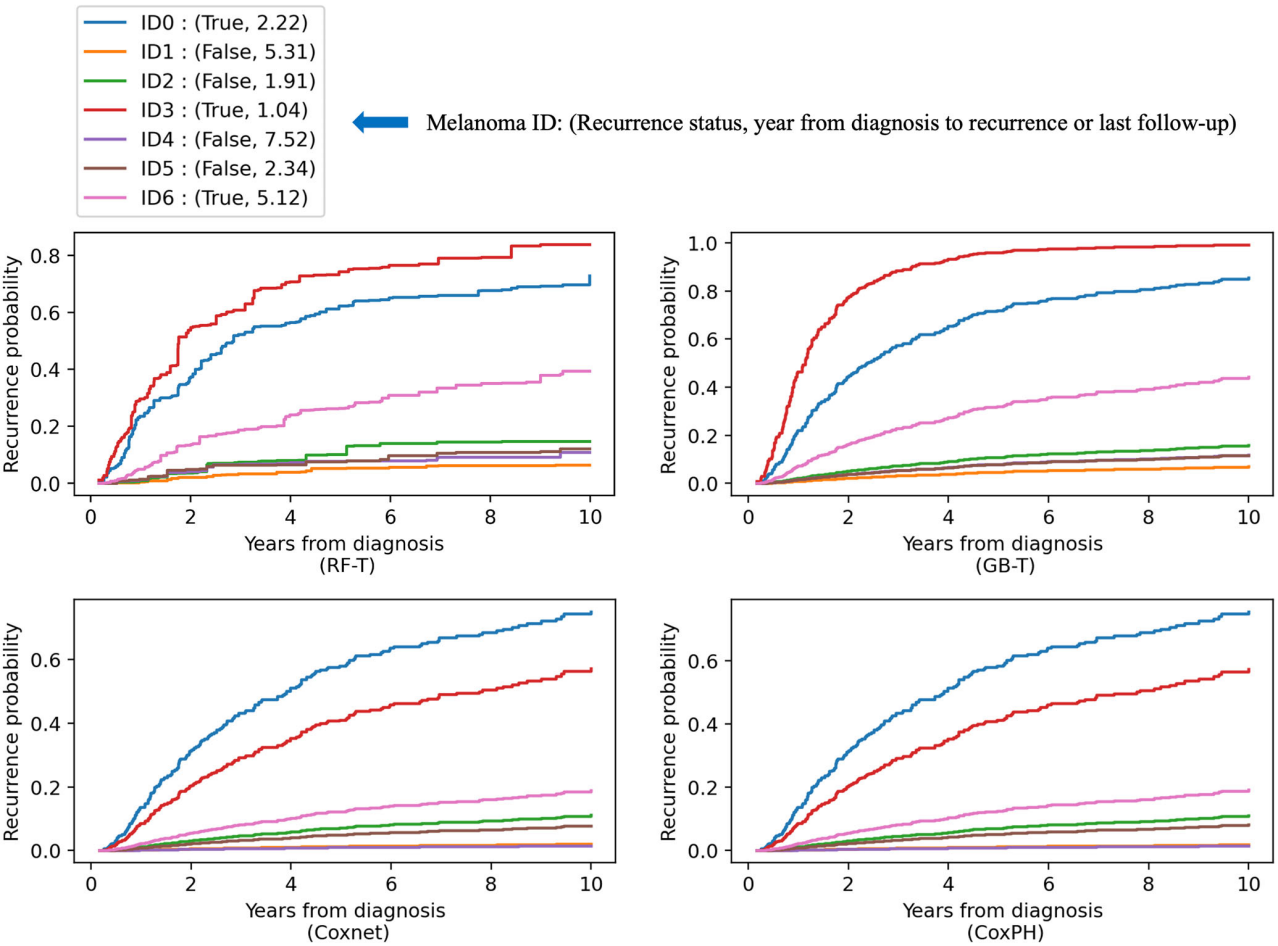
Recurrence probabilities for seven randomly selected melanomas in the external cohort (DFCI) predicted by the four time-to-event models trained by the MGB cohort. ID3 (red), ID0 (blue), and ID6 (pink) recurred with an increased duration from diagnosis of the primary melanoma to recurrence. The RF-T and GB-T models predicted them to have the highest recurrence probability proportional to their duration from diagnosis to recurrence. In the comparison of ID0 (recurrence) and ID5 (non-recurrence) where they had similar duration from diagnosis to recurrence or to last follow-up (ID0: 2.22, ID5: 2.34), all four models predicted ID0 to have a higher recurrence probability.

Recurrence probabilities for seven randomly selected melanomas from the original DFCI cohort predicted by the four time-to-event models trained by the original MGB cohort are presented in Fig. 6. Among the seven examples, ID3, ID0, and ID6 recurred with an increased duration from diagnosis of the primary melanoma to recurrence. The RF-T and GB-T models predicted them to have the highest recurrence probability proportional to their duration from diagnosis to recurrence.

### Feature importance in time-to-event recurrence risk prediction

**Fig. 7:**
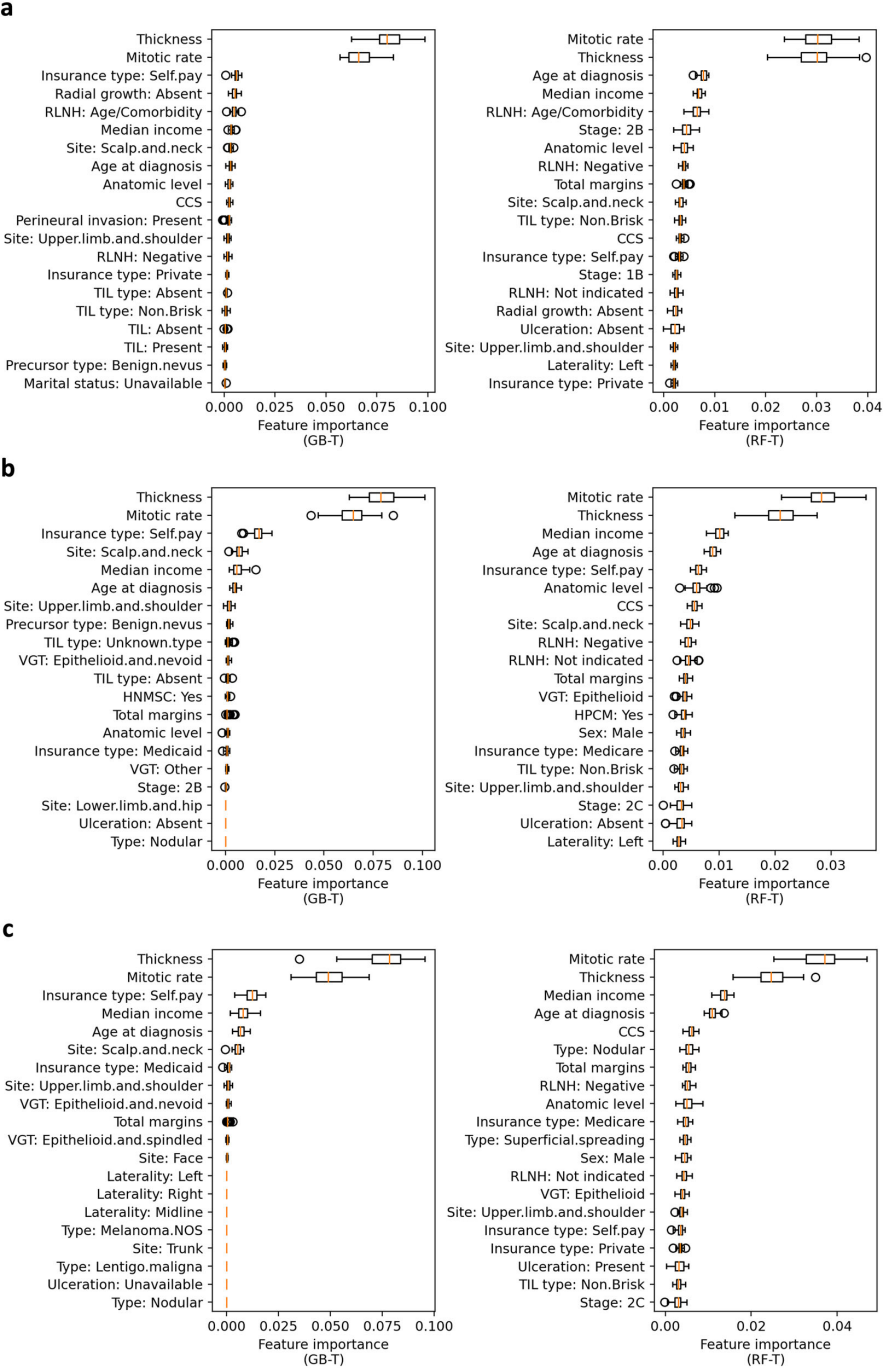
Feature importance in time-to-event recurrence prediction by RF-T and GB-T models. **a** The 20 most important features when experimenting on the original cohorts. **b** The 20 most important features when experimenting on the core complete cohorts: negative or not indicated regional lymph node histology, known mitotic rate, and known ulceration. **c** The 20 most important features when experimenting on the complete cohorts: negative or not indicated regional lymph node histology, known median income, and all tumor features available. The box extends from the first quartile to the third quartile of the feature importance values for each feature, with a line at the median. The whiskers extend from the box by 1.5x the interquartile range. Flier points are those past the end of the whiskers.

Feature importance in time-to-event prediction is presented in Fig. 7 and Supplementary 5. Results on the three types of cohorts were consistent, where Breslow thickness and mitotic rate were the two most important features. Additional important features included insurance type, age at diagnosis, median income, and absent radial growth phase. When experimenting with the case where anatomic level, Breslow thickness, and ulceration were removed and with the case where anatomic level and AJCC stage were removed (Supplementary Table 9 and Supplementary Fig. 6), overall consistent performances were achieved. In the first case, AJCC stage became one of the top three features by the GB-T and RF-T models. In the second case, ulceration became more important in both GB-T and RF-T models compared with the results when all extracted features were used.

### Sample size verification

We performed sample size calculations to ensure that our sample size was sufficient to evaluate the capacity of the machine-learning models. Using the observed true-positive rate of 0.79, the observed false-positive rate of 0.21, and a resulting standard difference of 1.2 in the internal validation, a sample size of 24 was required to achieve a power of 0.8 and a type I error rate of 0.05. As we had 215 recurrent melanomas in the MGB cohort and non-recurrent melanomas were randomly sampled to match the number of recurrent cases, the total number of samples was 430. With fivefold cross-validation, there were 86 testing samples in each experiment, which was sufficient for internal validation. Using the observed true-positive rate of 0.78, the observed false-positive rate of 0.22, and a resulting standard difference of 1.1 in the external validation, a sample size of 26 was required to achieve a power of 0.8 and a type I error rate of 0.05. As we had 95 recurrent melanomas in the DFCI cohort and non-recurrent melanomas were randomly sampled to match the number of recurrent cases, the total number of samples was 190. Thus, our models were adequately powered (>80%) to make predictions.

## Discussion

The increasing number of deaths associated with early-stage melanoma recurrence and the recently expanded indications for adjuvant immunotherapy in the management of early-stage melanomas highlight the need for prognostic tools to support clinicians in counseling patients and determining the optimal surveillance and risk stratification strategy in this population. We have performed the largest and most comprehensive study to date assessing the ability of machine-learning algorithms to predict early-stage melanoma recurrence using clinicopathologic features extracted from EHR data in a real-world clinical setting.

In the melanoma recurrence vs. non-recurrence classification, we were able to delineate melanomas with recurrence from non-recurrence. The GB models achieved the best performance in both internal (AUC: 0.845; PPV: 0.803) and external (AUC: 0.812; PPV:0.785) validations. These results demonstrate that, as previously shown with other primary malignancies^25^, we can reliably predict early-stage melanoma recurrence. A literature survey^25^ published recently reviewed the use of machine-learning techniques in predicting various outcomes related to cancer diagnosis and prognosis, including susceptibility, recurrence, and survival. Studies investigating cancer recurrence generally reported AUC in the range from 0.726 to 0.846^25^. The AUC values of these models have been shown to depend primarily on how much information is incorporated into the model, what type of information is used, and what specific outcomes are investigated. Our AUC values are consistent with the recent studies that investigated the recurrence of prostate, non-small cell lung, colorectal, and biliary cancers, with reported AUC ranging from 0.581 to 0.894^26–31^. The incorporation of more potentially predictive features tends to improve the performance of models. One study predicting oral cancer recurrence was able to achieve a reported accuracy of 0.917 after incorporating genomics-related data into the model^25,32^. Models based primarily on image analysis of digital pathology often reported high AUC values in the range from 0.861 to 0.993^33–35^. Based on the existing literature, it is reasonable to expect that by incorporating additional data, such as genomics and digital histopathology, our model performance would be significantly improved.

In the time-to-event recurrence prediction, the GB-T models achieved the best performance (internal time-dependent AUC: 0.853, concordance index: 0.820; external time-dependent AUC: 0.820, concordance index: 0.809), demonstrating that our models can achieve reliable performance in the time-to-event analysis. We expect that incorporating additional samples and relevant features, such as the histopathologic images and genomic data, will further improve the model performance.

In the recurrence group, 18% (55/310) of early-stage melanomas recurred after 5 years following the primary melanoma diagnosis, and among this population, 36% (20/55) recurred distantly (Stage IV at the time of recurrence). Current post-surgical monitoring guidelines for early-stage melanoma recurrence include serial skin examinations, regional lymph node examinations, and/or imaging modalities^36,37^. These recommendations and long-term surveillance follow-up vary depending on the stage of disease and individualized risk factors (i.e., family history)^36,37^. As demonstrated in Fig. 6, recurrence probabilities can differ significantly in a sample of 7 melanomas from our cohort over a 10-year time period depending on clinical and histopathologic features. The recurrence probability of melanoma ID04 (>0.8) was significantly higher as compared to melanoma ID01 (<0.1) at the 10-year follow-up mark. The time-to-event analysis provides an additional tool to support clinicians in the decision-making process of customizing surveillance and counseling patients diagnosed with early-stage primary melanomas.

We also assessed the impact of individual risk factors on machine-learning prediction of melanoma recurrence. These were ranked using feature importance in the prediction by each model, and the results provided further support for previously established risk factors in addition to identifying new trends that have not been reported previously. Breslow thickness has long been established as one of the most predictive features for melanoma recurrence^38^, and the results of this study support this finding. In our comprehensive feature model (including Breslow thickness, anatomic level, AJCC stage, and ulceration), Breslow thickness was one of the most important features both in the recurrence vs. non-recurrence classification by the RF and GB models and in the time-to-event recurrence prediction by the RF-T and GB-T, which typically yielded the most accurate prediction.

Currently, Breslow thickness and ulceration are the criteria used to determine the tumor (T) stage in early-stage disease^39^. While AJCC stage is another commonly cited risk factor for melanoma recurrence^12^, the stage was not in the top five most important features for recurrence prediction. This is likely due to how the machine-learning models account for collinearity. For example, when the model extracted predictive signals from Breslow thickness, it would not extract the same signals contained in AJCC stage. In addition, Breslow thickness was incorporated as a continuous variable, which may provide more detailed information as compared to the broad AJCC stage, leading to a higher feature ranking in our RF, GB, RF-T, and GB-T models. After performing sensitivity analyses by removing anatomic level, Breslow thickness, and ulceration (Supplementary Table 7 and Supplementary Fig. 4 for the binary recurrence classification, Supplementary Table 9 and Supplementary Fig. 6 for time-to-event prediction), AJCC stage was one of the two most important features by the GB, GB-T, and RF-T models and became a more important ranking feature in the RF model compared to the original cohorts. In particular, stage 1A was ranked as a higher predictive feature compared to other stages.

Mitotic rate was another critical feature in the discrimination of recurrence vs. non-recurrence by the RF and GB models and in the time-to-event prediction by the RF-T and GB-T models. Mitotic rate was the second most important feature in the MLP and LR models for the recurrence classification (Supplementary Fig. 3) and in the Coxnet and CoxPH models for the time-to-event prediction (Supplementary Fig. 5). Previous studies have identified tumor mitotic rate or presence of mitoses as a risk factor for recurrence, which our results support^10,40,41^. In the most recent AJCC melanoma staging system, mitotic rate is no longer a T1 staging criterion^14,39,42,43^. The most recent ASCO-SSO guidelines for sentinel lymph node biopsy also removed mitotic rate greater than 1 per mm^2^ as a high-risk factor for sentinel lymph node biopsy consideration, however mitotic rate can still be taken into account when considering a patient’s overall risk based on clinicopathologic features^43,44^. Previously, the mitotic rate was included in the 7^th^ edition of the AJCC staging criteria^13^. Per the guidelines, all melanomas with mitoses ≥1 mm^2^ were upstaged from 1 A to 1B.^13^, however this criterion was removed due to the poor inter-user reliability of mitotic rates^45^. Despite this, given how our models performed, with the mitotic rate being a more important feature than Breslow thickness in some machine-learning models, this feature should be increasingly evaluated in future studies and during consultations in clinical care. In our analyses, the mitotic rate was defined as a continuous variable, allowing the machine-learning algorithm to use detailed information for recurrence prediction, as compared to the AJCC 7th edition categories of <1 mm^2^ and ≥1 mm^2^ ^13^. In addition, the prior mitotic rate criterion only affected melanomas diagnosed as stages 1A and 1B. Our results show that mitotic rate can be broadly incorporated in the risk assessment for melanoma recurrence across all stages (1A, 1B, 2A, 2B, and 2C) of early-stage primary melanomas.

Finally, this study also included social determinants of health such as patient median income and insurance type. Median income was in the top ten predictive risk factors in our best performing models (RF, GB, RF-T, and GB-T), and the insurance type “self-pay” was in the top ten predictive risk factors in the GB, LR, and MLP models, and was in the top three predictive risk factors in the GB-T models. To investigate the reasons for our observations, we used a linear regression analysis to examine the association between thickness and patient demographics (sex, age at diagnosis, race, ethnicity, insurance type, median income, and marital status). We found that patients with Self-Pay and Medicaid insurance were at significantly greater risk for presenting with thicker melanomas at the time of diagnosis (Beta: 0.64, *p* < 0.001 for Self-Pay; 1.1, *p* = 0.025 for Medicaid). The findings are consistent with prior literature identifying delays in primary melanoma diagnosis and increased diagnosis of advanced-stage melanoma among uninsured or publicly insured patients. In one study, individuals who are underinsured have been found to present with thicker tumors (OR: 2.19) compared with non-Medicaid insured patients^46^. Similarly, Medicaid patients were found to also present with thicker tumors at diagnosis compared to those not on Medicaid coverage (OR: 2.37)^47^. Since Medicaid is the primary insurance coverage for low-income individuals and self-pay patients are likely underinsured individuals, our results demonstrate socioeconomic disparities in the timeliness of melanoma diagnosis. These SES risk factors are also surrogate markers that affect melanoma care, from diagnosis to treatment and surveillance. Medicaid patients experience delays in surgical treatment of primary melanomas by 6 weeks^48^. Although, it is unknown whether delay in surgical treatment affects mortality, timing of melanoma treatment should be investigated in future studies to assess associations between risk of recurrence and timing of recurrence.

The overall results of our feature importance ranking remained consistent after sensitivity analyses where only melanomas with negative or not indicated regional lymph node histology, and melanomas without no missing values for ulceration/mitotic rate were included. Breslow thickness and mitotic rate were consistently the two most important features for classification by RF and GB binary classification models (Fig. 5) and for time-to-event prediction by RF-T and GB-T models (Fig. 7). Median income and the insurance type, self-pay, remained in the top five important features classification by RF and GB binary classification models and for time-to-event prediction by RF-T and GB-T models. We also observed only a mild decrease in AUC (0.812 to 0.809 for classification; 0.820 to 0.804 for time-to-event prediction) in these more restricted settings. This is expected as our sample size was reduced, however, the consistent results demonstrate the robustness of the GB and GB-T models in the binary classification and time-to-event prediction of melanoma recurrence. Despite limitations with missing clinical or histology features, our sensitivity analyses show our models yield robust results.

This study was also limited by its retrospective nature. However, with 1,720 included melanomas, our study represents an improvement in sample size and predictive power compared to many prior studies investigating risk factors associated with melanoma recurrence. Additionally, the mitotic rate variable in this study was extracted from pathology reports, which is subject to high levels of interobserver variation. Future investigations incorporating automatic mitotic feature detection from H&E whole slide images using similar deep learning approaches to other cancers (e.g., breast cancer)^49,50^ are necessary to evaluate the performance of a more objective mitotic rate variable in predicting melanoma recurrence. Despite this limitation, mitotic rate was nevertheless consistently selected as a top performing feature in our models. It is also possible that some of the non-recurrent melanomas included in our study experienced recurrence after the end of study follow-up (false non-recurrence). However, this is likely to affect a minority of the population since (1) more than 80% of our study population experienced recurrence within 5 years (and more than 90% within 7 years), and (2) we specifically selected our control population to have a minimum of 5 years of follow-up in the binary melanoma recurrence classification tasks. Furthermore, a sensitivity analysis was performed to compare the performance of binary classification when the criteria for non-recurrent melanomas was extended to 7 years. The results of the sensitivity analysis (Table 2) show consistent performance (AUC: 0.827) compared to the primary models with criteria of at least 5 years of follow-up. Overall, this study also provided longer follow-up than available in previous investigations, further decreasing the possibility of misclassification of non-recurrent melanomas. There were no minimum follow-up constraints for non-recurrent melanomas in the time-to-event analysis, and therefore the risk of false non-recurrence is not applicable to this analysis. In the time-to-event recurrence prediction, the censoring status for non-recurrences was classified as either dead or censored. The time to event for recurrences was defined as the duration from primary melanoma diagnosis to recurrence. The time to event for non-recurrences was defined as the duration from primary melanoma diagnosis to the date of death or date of censoring.

In summary, we demonstrate consistent and reliable performance of machine-learning models for predicting melanoma recurrence in the largest cohort of patients diagnosed with early-stage melanomas to date. We delineate the most significant features for recurrence prediction, which include Breslow thickness, mitotic rate, AJCC stage, median income, insurance type, and age at diagnosis. Overall, our study provides important insights for clinicians to counsel patients on the contributions of individualized risk factors for early-stage melanoma recurrence. However, despite the incorporation of a comprehensive array of over 36 demographic, clinical, and histopathologic features, there is a plateau in model performance for the recurrence classification (AUC: 0.812) and for the time-to-event recurrence prediction (time-dependent AUC: 0.820). The predictive capabilities of these models can benefit from the incorporation of additional features, including digital histopathology images, genomics data, and novel tumor biomarkers. Nevertheless, our presented models may be deployed clinically to aid in the identification of high-risk early-stage melanoma patients that may benefit from increased surveillance or adjuvant immunotherapy.

## Methods

This study was conducted to determine the capabilities of machine-learning algorithms to predict early-stage melanoma recurrence using patient demographics, medical history, and melanoma tumor characteristics. We performed two types of prediction by using nine machine-learning algorithms: (1) melanoma recurrence classification; (2) time-to-event melanoma recurrence risk prediction. Models were all validated internally and externally. First, we used the stratified fivefold cross-validation on the MGB cohort (internal validation). Second, we trained models using the MGB cohort and evaluated them independently on the DFCI cohort (external validation) (Supplementary Fig. 1).

### Materials

Patients diagnosed with Stage I or Stage II melanoma at MGB and DFCI between January 2000 and February 2020 were included in this study. Melanoma histology (confirmed by ICD-O-3 codes), diagnosis date of the primary melanoma, and recurrence date were extracted from the cancer registrars at both institutions. Cutaneous melanomas with no evidence of metastasis at the time of diagnosis and of the following histological types were included: lentigo maligna, nodular, superficial spreading, and melanoma NOS (not otherwise specified). Acral, mucosal, and uveal melanomas were excluded from the study. The Mass General Brigham Institutional Review Board (IRB) approved the study (Protocol # 2020P002179). The need for consent was waived by the IRB as the study meets the criteria for exemption 45 CFR 46.104(d)(#). Secondary research for which consent is not required if the research involves only information collection and analysis involving the investigator’s use of identifiable health information when that use is regulated under 45 CFR parts 160 and 164, subparts A and E.

The Research Patient Data Registry (RPDR)^51^ and the Enterprise Data Warehouse^52^ (EDW) are two institutional clinical databases at MGB and DFCI, which were used to extract clinical and medical history information. The RPDR contains multimodal clinical information, including basic demographics, International Classification of Diseases (ICD) codes, and lab results. The EDW provides detailed documentation of medication administrations. For this study, we extracted the following variables from RPDR: date of birth, sex, race, ethnicity, insurance type, marital status, zip code, date of death or last follow-up. Median income was extracted from the U.S. Census data based on the patient’s zip code^53^. ICD codes from all visits of a patient before the diagnosis of early-stage melanoma were used to calculate Charlson Comorbidity score^54^ (CCS) and to extract medical history features (Supplementary Table 1). The ICD codes used to identify non-melanoma skin cancer, benign neoplasms of skin, cutaneous autoimmune diseases, and other systemic autoimmune diseases were specified in Supplementary Tables 10–12. Mortality information was additionally ascertained via the institutional cancer registrars using linkage with obituary databases and the National Death Index (NDI). For patients who did not have known mortality information, their last encounter with the system was considered their censoring date.

We obtained data for collecting features of interest (Supplementary Table 1) using established protocols for electronic data extraction from institutional clinical databases. For all features with incomplete information, rigorous manual chart review and data extraction were performed. Manual chart reviews by two independent reviewers were conducted to ascertain the recurrence status (recurrence vs. non-recurrence) and recurrence type (locoregional recurrence vs. distant recurrence). All melanomas that were Stage IV at the time of recurrence, based on the American Joint Committee on Cancer (AJCC) 8th edition staging guidelines^39^, were labeled as having a distant recurrence. All recurrent melanomas without distant metastases were labeled as having a locoregional recurrence. In classification tasks, melanomas that did not recur and were followed up at least 5 years were labeled as “non-recurrence”. If a patient died without melanoma recurrence and the follow-up duration was less than 5 years, the patient was not included in the binary classification tasks. In the time-to-event prediction tasks, all melanomas without recurrence were labeled as “non-recurrence” regardless of the follow-up duration. The time to event is the duration from diagnosis to recurrence if the melanoma recurred; otherwise, the time to event is the duration from diagnosis to date of death or last follow-up.

Figure 1 shows the flow diagram of how the study population was obtained. The MGB population was utilized for model development and the DFCI population was utilized for external validation of the model. In total, 36 features were extracted for inclusion in our registry (Supplementary Table 1), which can be categorized into three groups: demographics, medical history, and tumor characteristics. Specifically, seven demographic features include age at diagnosis, sex, race, ethnicity, median income, insurance type, and marital status. Medical history includes seven features: Charlson comorbidity score (CCS), history of prior cutaneous melanoma (HPCM), history of non-melanoma skin cancer (HNMSC), history of situ or benign neoplasms of skin (HSBN), history of other malignancy (HOM), history of cutaneous autoimmune disease (HCAID), and history of systemic autoimmune disease (HSAID). Tumor characteristics consist of 22 features: histological type, tumor site, AJCC stage at diagnosis, Breslow thickness, anatomic level, mitotic rate, ulceration, laterality, tumor infiltrating lymphocytes (TIL), tumor infiltrating lymphocytes type, precursor lesion, precursor type, radial growth phase, vertical growth phase, vertical growth type (VGT), microsatellites, regression, lymphovascular invasion (LVI), perineural invasion, total surgical margins of excision(s), check if the surgical margins of the excision(s) met management guidelines (margin check)^36,55^, and regional lymph node histology (RLNH). All histopathologic features included in this study were collected from treatment-naïve biopsies of primary melanomas at the time of melanoma diagnosis and the included tumors were all early-stage at diagnosis. Therefore, no patients included in our study have received treatment with systemic therapy for their primary early-stage melanomas.

Based on clinical guidelines for melanoma, all melanomas with tumor categories above T1b (Breslow thickness >1.00 mm) are recommended to undergo a sentinel lymph node biopsy (SLNB)^44^. SLNB was not recommended for patients diagnosed with primary melanomas that are <0.8 mm thick, non-ulcerated lesions (T1a)^44^. Patients diagnosed with primary melanomas that are 0.8 to 1.0 mm thick or are <0.8 mm thick, ulcerated lesions (T1b) may be offered a SLNB after a clinical discussion of the risks and benefits of the procedure with their provider^44^. As a result, patients with stage IA melanoma were not recommended to undergo a SLNB due to the low overall rate of SLNB positivity in this population. Additionally, in real-world clinical settings, patients may defer the SLNB due to various reasons, e.g., comorbidity, frailty, unwillingness to undergo an additional invasive procedure, etc. Thus, we detailed the variable regional lymph node histology (RLNH) incorporating this complexity of SLNB in a real-world clinical setting, which includes the following values: 0. all nodes negative; 1. not indicated; 2. not performed due to age or comorbidity; 3. not performed due to an unknown reason; 4. deferred by the patient.

To reduce the degree of omission for many included synoptic features, we expanded our manual phenotyping efforts, including reviews of manually scanned pathology reports uploaded into the electronic medical records and reviews of all available clinical notes in the electronic medical records, and incorporated input from dermatopathologists with institutional experience in synoptic feature reporting across time. Given that our data was extracted from real-world electronic medical records (non-clinical trial settings), some variables, like precursor lesion and perineural invasion, may not be included in the pathology report if they were not identified in the biopsy by the pathologist. As a result, and with input from dermatopathologists with expert knowledge of the reporting schema at our institutions, precursor lesion, precursor type, microsatellites, regression, lymphovascular invasion, and perineural invasion were assumed absent if not listed as present in the pathology report. All melanomas without a pathology report available and melanomas with positive microscopic satellites were excluded from this study.

Categorical features were converted by one-hot encoding (Supplementary Table 2). For missing continuous features (Supplementary Table 1), we assigned the median values of the samples to individuals, including median income, Charlson comorbidity score, mitotic rate, and total surgical margins.

### Machine-learning methods for binary recurrence classification

We compared the classification performances of five classic supervised machine-learning algorithms using the extracted clinicopathologic features. The algorithms include Support Vector Machine (SVM)^56^, Gradient Boosting (GB)^57,58^, Random Forest (RF)^59^, Logistic Regression (LR)^60^, and Multi-layer Perceptron (MLP)^61,62^. Model parameters were optimized by cross-validated grid-search over a parameter grid on the MGB cohort. The kernel of SVM was the radial basis function. The GB had 100 estimators. The L2 regularization was used for LR to reduce overfitting. The MLP was performed with a logistic activation function. Model performance was evaluated based on the area under the receiver operating characteristic curve (AUC), positive predictive value (PPV), sensitivity, specificity, and accuracy. Mean and 95% confidence intervals were reported. All experiments were implemented using scikit-learn Python library^24^.

#### Binary melanoma recurrence classification

For our primary outcome, we evaluated the ability of machine-learning models to predict the recurrence (recurrence vs. non-recurrence). Stratified fivefold cross-validation was applied in the internal validation, which preserves the percentage of samples for each class in each fold. In the external validation, the entire MGB cohort was used for training models and the DFCI cohort was left out for independently evaluating the models. Each experiment was repeated 50 times, and each time non-recurrent melanomas were randomly sampled to match the number of recurrent cases (Supplementary Fig. 1a). We further investigated the predictive features used in the recurrence prediction by conducting permutation importance^24^. The permutation importance for feature evaluation was repeated for 50 times and the AUC was used for scoring.

#### Sensitivity analyses

Ensuring sentinel lymph node biopsy negativity is important to be able to exclude possibility of nodal involvement at time of diagnosis. However, given that our data was extracted from EHRs in a real-world clinical setting and to remain consistent with clinical guidelines, we didn’t exclude patients who didn’t perform sentinel lymph node biopsy at time of diagnosis in our main analysis. In the sensitivity analysis, we excluded the melanomas for which the sentinel lymph node biopsy was indicated but not performed, ulceration was missing, or mitotic rate was unknown. We also conducted experiments on a complete data for which median income and all tumor features were without missing values. In our main analysis, a minimum of 5-year follow-up was used to ensure sufficient time to observe a recurrence in the non-recurrence population. Though the melanoma recurrence rate decreases significantly after 5 years (Fig. 2), the minimum 5-year follow-up duration may not have captured all recurrences. Therefore, we further performed binary recurrence classification experiments when the minimum follow-up duration for the non-recurrent melanomas was 7 years.

### Machine-learning methods for time-to-event recurrence prediction

Melanoma recurrence is a time-to-event outcome. It is important to predict the risk probability as time goes by. We compared the time-to-event prediction performances of four supervised machine-learning algorithms using the extracted clinicopathologic features. The algorithms include GradientBoostingSurvivalAnalysis^63^ (GB-T), RandomSurvivalForest^64^ (RF-T), CoxnetSurvivalAnalysis^65^ (Coxnet), and CoxPHSurvivalAnalysis^66^ (CoxPH). Similar to the classification tasks, model parameters were optimized by cross-validated grid-search over a parameter grid on the MGB cohort. Model performance was evaluated based on the time-dependent AUC^67^ and concordance index for right-censored data^68^. Each experiment was repeated 50 times. Mean and 95% confidence intervals were reported. All experiments were implemented by using scikit-survival Python library^69^.

We also investigated the predictive features by conducting permutation importance^24^. The permutation importance for feature evaluation was repeated for 50 times and the concordance index was used for scoring. Sensitivity analysis on cohorts with negative or not indicated sentinel lymph node biopsy and with available tumor features was also conducted.

### Statistical methods

The minimum sample size at a power of 0.8 and a type I error rate of 0.05 were calculated to ensure that our study sample size was sufficient to evaluate the capacity of models for predicting melanoma recurrence^70^. To compare groups, we used Pearson’s Chi-squared test or Fisher’s exact test for categorical variables, and the *t*-test or Kruskal–Wallis test for continuous variables, provided by the stats package in R version 4.1.0^71^.

### Reporting summary

Further information on research design is available in the Nature Research Reporting Summary linked to this article.

## Supplementary information

Supp

REPORTING SUMMARY

## Data Availability

All relevant data are available from the corresponding author: Yevgeniy R. Semenov. All summary data supporting the findings of this study are available within the article and/or its supplementary materials. The patient data generated for this study can only be shared per specific institutional review board (IRB) requirements. Upon a request to the corresponding author, a data-sharing agreement can be initiated following institution-specific guidelines.

## Code availability

Data collection and analyses were performed in R 4.2.1, Python 3.8.12, NumPy 1.20.2, scikit-learn 0.24.1, and scikit-survival 0.17.2. The code is available at https://github.com/SemenovLab/early-stage-melanoma-recurrence-prediction.

## References

[1] CDC. *Melanoma Incidence and Mortality, United States—2012–2016* (Centers for Disease Control and Prevention, US Department of Health and Human Services, 2012–2016).

[2] Bajaj S (2020). Melanoma prognosis: accuracy of the American Joint Committee on Cancer Staging Manual Eighth Edition. J. Natl Cancer Inst..

[3] Freeman M, Laks S (2019). Surveillance imaging for metastasis in high-risk melanoma: importance in individualized patient care and survivorship. Melanoma Manag..

[4] FDA. *FDA Approves Pembrolizumab for Adjuvant Treatment of Stage IIB or IIC Melanoma* (United States Food and Drug Administration, 2021).

[5] Tang K (2022). Association of cutaneous immune-related adverse events with increased survival in patients treated with anti-programmed cell death 1 and anti-programmed cell death ligand 1 therapy. JAMA Dermatol.

[6] Wongvibulsin S (2022). Epidemiology and risk factors for the development of cutaneous toxicities in patients treated with immune-checkpoint inhibitors: A United States population-level analysis. J. Am. Acad. Dermatol..

[7] Patrinely JR (2021). Chronic immune-related adverse events following adjuvant anti-PD-1 therapy for high-risk resected melanoma. JAMA Oncol..

[8] von Schuckmann LA (2019). Risk of melanoma recurrence after diagnosis of a high-risk primary tumor. JAMA Dermatol..

[9] Cho SI (2019). Local recurrence and metastasis in patients with malignant melanomas after surgery: A single-center analysis of 202 patients in South Korea. PLoS ONE.

[10] Matheson JA (2017). Prospective evaluation of prognostic indicators for early recurrence of cutaneous melanoma. Melanoma Res..

[11] Urist MM (1985). The influence of surgical margins and prognostic factors predicting the risk of local recurrence in 3445 patients with primary cutaneous melanoma. Cancer.

[12] Feigelson HS (2019). Melanoma incidence, recurrence, and mortality in an integrated healthcare system: a retrospective cohort study. Cancer Med..

[13] Balch CM (2009). Final version of 2009 AJCC melanoma staging and classification. J. Clin. Oncol..

[14] Keung EZ, Gershenwald JE (2018). The eighth edition American Joint Committee on Cancer (AJCC) melanoma staging system: implications for melanoma treatment and care. Expert Rev. Anticancer Ther..

[15] Callender GG (2011). Prognostic implications of anatomic location of primary cutaneous melanoma of 1 mm or thicker. Am. J. Surg..

[16] Shaikh, W. R., et al. Melanoma thickness and survival trends in the United States, 1989 to 2009. *J. Natl Cancer Inst*. **108**, djv294 (2016). 10.1093/jnci/djv294 PMC4857148 26563354

[17] El Sharouni, M. A. et al. The progressive relationship between increasing Breslow thickness and decreasing survival is lost in patients with ultrathick melanomas (>/=15 mm in thickness). *J. Am. Acad. Dermatol.* **87**, 298–305 (2022). 10.1016/j.jaad.2022.01.040 35121073

[18] Azzola MF (2003). Tumor mitotic rate is a more powerful prognostic indicator than ulceration in patients with primary cutaneous melanoma: an analysis of 3661 patients from a single center. Cancer.

[19] Helgadottir H (2015). CDKN2a mutation-negative melanoma families have increased risk exclusively for skin cancers but not for other malignancies. Int. J. Cancer.

[20] Leung B (2022). 658 Clinical and histopathologic risk factors for early-stage melanoma recurrence. J. Investig. Dermatol..

[21] Abdullah Alfayez A, Kunz H, Grace Lai A (2021). Predicting the risk of cancer in adults using supervised machine learning: a scoping review. BMJ Open.

[22] Richter, A. N. & Khoshgoftaar, T. M. Melanoma risk prediction with structured electronic health records. in *Proceedings of the 2018 ACM International Conference on Bioinformatics, Computational Biology, and Health Informatics* (Association for Computing Machinery, Washington, DC, USA, 2018).

[23] Hornbrook MC (2017). Early colorectal cancer detected by machine learning model using gender, age, and complete blood count data. Dig. Dis. Sci..

[24] Pedregosa F (2011). Scikit-learn: machine learning in Python. J. Mach. Learn. Res..

[25] Kourou K, Exarchos TP, Exarchos KP, Karamouzis MV, Fotiadis DI (2015). Machine learning applications in cancer prognosis and prediction. Comput. Struct. Biotechnol. J..

[26] Tan YG (2022). Incorporating artificial intelligence in urology: Supervised machine learning algorithms demonstrate comparative advantage over nomograms in predicting biochemical recurrence after prostatectomy. Prostate.

[27] Beinecke JM (2022). Evaluation of machine learning strategies for imaging confirmed prostate cancer recurrence prediction on electronic health records. Comput. Biol. Med..

[28] Hindocha S (2022). A comparison of machine learning methods for predicting recurrence and death after curative-intent radiotherapy for non-small cell lung cancer: Development and validation of multivariable clinical prediction models. EBioMedicine.

[29] Leonard G (2022). Machine learning improves prediction over logistic regression on resected colon cancer patients. J. Surg. Res..

[30] Jolissaint, J. S. et al. Machine learning radiomics can predict early liver recurrence after resection of intrahepatic cholangiocarcinoma. *HPB (Oxford)* **24**, 1341–1350 (2022). 10.1016/j.hpb.2022.02.004 PMC9355916 35283010

[31] Chen, P. C. et al. A prediction model for tumor recurrence in stage II-III colorectal cancer patients: from a machine learning model to genomic profiling. *Biomedicines* **10**, 340 (2022). 10.3390/biomedicines10020340 PMC8961774 35203549

[32] Exarchos KP, Goletsis Y, Fotiadis DI (2012). Multiparametric decision support system for the prediction of oral cancer reoccurrence. IEEE Trans. Inf. Technol. Biomed..

[33] Marostica E (2021). Development of a histopathology informatics pipeline for classification and prediction of clinical outcomes in subtypes of renal cell carcinoma. Clin. Cancer Res..

[34] Hayashi, K. et al. Prediction of recurrence pattern of pancreatic cancer post-pancreatic surgery using histology-based supervised machine learning algorithms: a single-center retrospective study. *Ann. Surg. Oncol.*10.1245/s10434-022-11471-x (2022). 10.1245/s10434-022-11471-x 35230581

[35] Wan G (2022). 649 CNN-based histopathology image analysis for early-stage melanoma recurrence. J. Investig. Dermatol..

[36] Swetter SM (2019). Guidelines of care for the management of primary cutaneous melanoma. J. Am. Acad. Dermatol.

[37] Swetter SM (2021). NCCN guidelines(R) insights: melanoma: cutaneous, version 2.2021. J. Natl Compr. Canc Netw..

[38] Lyth J, Falk M, Maroti M, Eriksson H, Ingvar C (2017). Prognostic risk factors of first recurrence in patients with primary stages I-II cutaneous malignant melanoma—from the population-based Swedish melanoma register. J. Eur. Acad. Dermatol. Venereol..

[39] Gershenwald JE, Scolyer RA (2018). Melanoma staging: American Joint Committee on Cancer (AJCC) 8th edition and beyond. Ann. Surg. Oncol..

[40] Laks S (2017). Tumor mitotic rate and association with recurrence in sentinel lymph node negative stage II melanoma patients. Am. Surg..

[41] Tas F, Erturk K (2022). Different mitotic rates are associated with different prognostic factors, relapses, and survival rates in melanoma. Int. J. Dermatol..

[42] Gershenwald JE (2017). Melanoma staging: evidence-based changes in the American Joint Committee on Cancer eighth edition cancer staging manual. CA Cancer J. Clin..

[43] Pathak, S. & Zito, P. M. Clinical guidelines for the staging, diagnosis, and management of cutaneous malignant melanoma. in *StatPearls* (Treasure Island (FL), 2022). 34283515

[44] Wong SL (2018). Sentinel lymph node biopsy and management of regional lymph nodes in melanoma: American Society of Clinical Oncology and Society of Surgical Oncology Clinical Practice Guideline Update. J. Clin. Oncol..

[45] Garbe C (2016). Mitotic rate in primary melanoma: interobserver and intraobserver reliability, analyzed using H&E sections and immunohistochemistry. J. Dtsch Dermatol Ges..

[46] Cortez JL, Vasquez J, Wei ML (2021). The impact of demographics, socioeconomics, and health care access on melanoma outcomes. J. Am. Acad. Dermatol..

[47] Pollitt RA, Clarke CA, Shema SJ, Swetter SM (2008). California Medicaid enrollment and melanoma stage at diagnosis: a population-based study. Am. J. Prev. Med..

[48] Adamson AS, Zhou L, Baggett CD, Thomas NE, Meyer AM (2017). Association of delays in surgery for melanoma with insurance type. JAMA Dermatol.

[49] Paeng, K., Hwang, S., Park, S. & Kim, M. A unified framework for tumor proliferation score prediction in breast histopathology. 231–239 (Springer International Publishing, Cham, 2017).

[50] Alom MZ, Aspiras T, Taha TM, Bowen TJ, Asari VK (2020). MitosisNet: end-to-end mitotic cell detection by multi-task learning. IEEE Access.

[51] Nalichowski, R., Keogh, D., Chueh, H. C. & Murphy, S. N. Calculating the benefits of a Research Patient Data Repository. *AMIA Annu. Symp. Proc*. **2006**, 1044 (2006). PMC1839563 17238663

[52] Format. *The Enterprise Data Warehouse (EDW): Creating the Foundation for Effective Healthcare Improvement Analytics* (Health Catalyst, 2015).

[53] Bureau, U. S. C. *Selected Income Characteristics, 2006–2020 American Community Survey 5-year Estimates* (United States Census Bureau, 2020).

[54] Roffman CE, Buchanan J, Allison GT (2016). Charlson comorbidities index. J. Physiother..

[55] NCCN. *Clinical Practice Guidelines in Oncology: Melanoma: Cutaneous* (Network, N.C.C.) https://www.nccn.org/professionals/physician_gls/pdf/cutaneous_melanoma.pdf (2022).

[56] Vapnik VN (1999). An overview of statistical learning theory. IEEE Trans. Neural Netw..

[57] Friedman JH (2002). Stochastic gradient boosting. Comput. Stat. Data Anal..

[58] Friedman JH (2001). Greedy function approximation: a gradient boosting machine. Ann. Stat..

[59] Breiman L (2001). Random Forests. Mach. Learn..

[60] Kleinbaum, D. G., Dietz, K., Gail, M., Klein, M. & Klein, M. *Logistic Regression* (Springer, 2002).

[61] Hinton, G. I. in *Machine Learning: an Artificial Intelligence Approach Volume III* 555Б─⌠610 (Morgan Kaufmann Publishers Inc., 1990).

[62] Glorot, X. & Bengio, Y. *Understanding the difficulty of training deep feedforward neural networks*. Vol. 9 (eds. Teh, Y. W. & Titterington, D. M.) 249–256 (JMLR.org, 2010).

[63] Hothorn T, Buhlmann P, Dudoit S, Molinaro A, van der Laan MJ (2006). Survival ensembles. Biostatistics.

[64] Hemant I, Udaya BK, Eugene HB, Michael SL (2008). Random survival forests. Ann. Appl. Stat..

[65] Simon N, Friedman J, Hastie T, Tibshirani R (2011). Regularization paths for Cox’s proportional hazards model via coordinate descent. J. Stat. Softw..

[66] Efron B (1977). The efficiency of Cox’s likelihood function for censored data. J. Am. Stat. Assoc..

[67] Lambert J, Chevret S (2016). Summary measure of discrimination in survival models based on cumulative/dynamic time-dependent ROC curves. Stat. Methods Med. Res..

[68] Harrell FE, Lee KL, Mark DB (1996). Multivariable prognostic models: issues in developing models, evaluating assumptions and adequacy, and measuring and reducing errors. Stat. Med..

[69] Pölsterl S (2020). scikit-survival: a library for time-to-event analysis built on top of scikit-learn. J. Mach. Learn. Res..

[70] Jones SR, Carley S, Harrison M (2003). An introduction to power and sample size estimation. Emerg. Med. J..

[71] Team, R.C. *R: A Language and Environment for Statistical Computing* (R Foundation for Statistical Computing, Vienna, Austria, 2018).

