# Supplementary material for: Prediction of early-stage melanoma recurrence using clinical and histopathologic features
Source: NPJ Precis Oncol. 2022 Oct 31;6:79. doi: 10.1038/s41698-022-00321-4 (PMC9622809; doi:10.1038/s41698-022-00321-4)
Supplement: Supplementary file 1 — Supp [file 41698_2022_321_MOESM1_ESM.pdf]

Prediction of Early-Stage Melanoma Recurrence Using Clinical and Histopathologic Features (Supplementary Material)

Supplementary Figure 1. Validation design.

a. Validation design for classification tasks: non-recurrences are randomly sampled to match the number of recurrences in each run of machine learning algorithms.

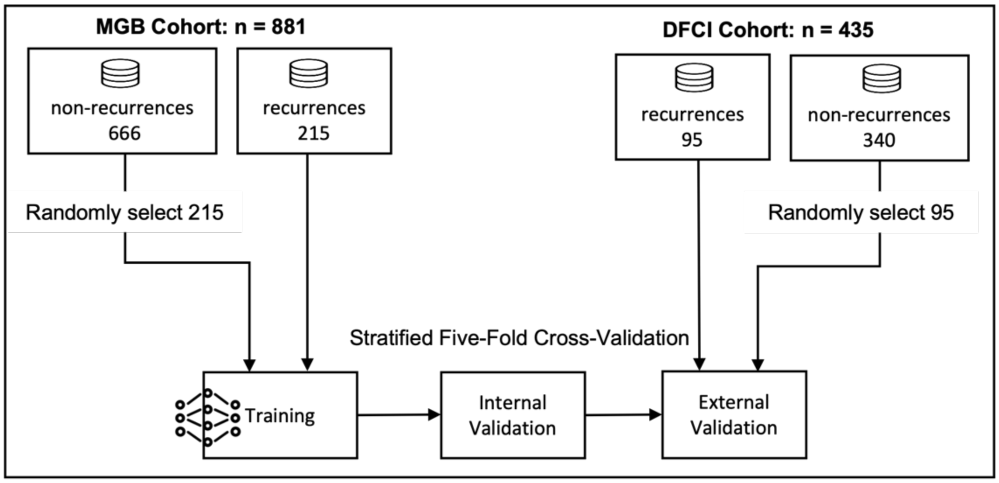

b. Validation design for time-to-event prediction tasks: non-recurrences are best matched to recurrences (3:1) in terms of follow-up duration in each cohort.

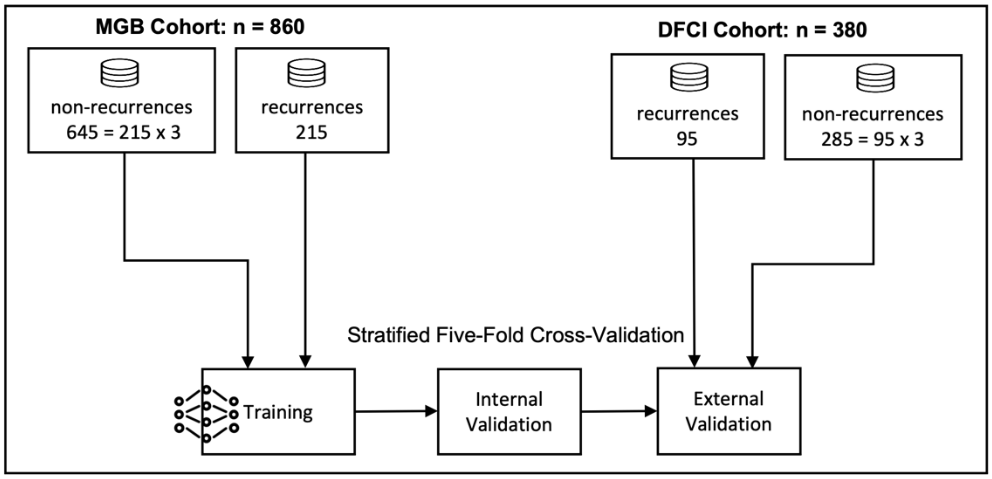

Supplementary Table 1. Clinicopathologic features to be assessed for early-stage melanoma recurrence prediction.

Index	Feature	Format	Count of missing values in the recurrence group (n=310)	Count of missing values in the non-recurrence Group (n=1,410)
Demographics				
1	Sex	Binary	0	0
2	Race	Categorical	6 (1.9%)	11 (0.8%)
3	Ethnicity	Categorical	5 (1.6%)	18 (1.3%)
4	Median income	Numerical	6 (1.9%)	21 (1.5%)
5	Insurance type	Categorical	0	0
6	Marital status	Categorical	8 (2.6%)	33 (2.3%)
7	Age at diagnosis	Numerical	0	0
Medical history				
1	History of prior cutaneous melanoma (HPCM) [a]	Binary	0	0
2	Charlson comorbidity score (CCS) [b]	Numerical	111 (35.8%)	302 (21.4%)
3	History of non-melanoma skin cancer (HNMSC) [b, c]	Categorical	above	above
4	History of situ or benign neoplasm of skin (HSBN) [b, c]	Categorical	above	above
5	History of other malignancy (HOM) [b]	Categorical	above	above
6	History of cutaneous autoimmune disease (HCAID) [b, c]	Categorical	above	above
7	History of systemic autoimmune disease (HSAID) [b, c]	Categorical	above	above
Tumor characteristics				
1	Histological type	Categorical	0	0
2	Tumor site	Categorical	0	0
3	AJCC stage [d]	Categorical	0	0
4	Breslow thickness	Numerical	0	0
5	Anatomic level	Numerical	0	0
6	Laterality	Categorical	0	0
7	Regional Lymph Node Histology (RLNH)	Categorical	Not performed with unknown reason: 12 (3.9%)<br>Not Performed due to age or comorbidity: 59 (19.0%)<br>Deferred by patient: 9 (2.9%)	Not performed with unknown reason: 30 (2.1%)<br>Not Performed due to age or comorbidity: 30 (2.1%)<br>Deferred by patient: 5 (0.4%)
8	Ulceration	Categorical	1 (0.3%)	4 (0.3%)
9	Mitotic rate	Numerical	14 (4.5%)	215 (15.2%)
10	Total excisional surgical margins (Total margins)	Numerical	34 (11.0%)	201 (14.3%)
11	Check if meeting margin requirements (Margin check)	Categorical	35 (11.3%)	204 (14.5%)

12	Tumor infiltrating lymphocytes (TIL)	Categorical	37 (11.9%)	375 (26.6%)
13	TIL type [d]	Categorical	37 (11.9%)	375 (26.6%)<br>Unknown type: 7 (0.5%)
14	Radial growth	Categorical	29 (9.4%)	111 (7.9%)
15	Vertical growth	Categorical	20 (6.5%)	150 (10.6%)
16	Vertical growth type (VGT)	Categorical	20 (6.5%)<br>Unknown type: 20 (6.5%)	150 (10.6%)<br>Unknown type: 92 (6.5%)
17	Precursor lesion [e]	Categorical	0	0
18	Precursor type [e]	Categorical	0	Unknown type: 15 (1.1%)
19	Microsatellites [e]	Categorical	0	0
20	Regression [e]	Categorical	0	0
21	Lymphovascular invasion (LVI) [e]	Categorical	0	0
22	Perineural invasion [e]	Categorical	0	0

[a] Manual chart review was conducted to extract this feature.

[b] All International Classification of Diseases (ICD) codes before the primary melanoma diagnosis were used to extract these features. There are 413 patients whose first visit to the healthcare system was due to melanoma care.

[c] The ICD codes used to extract these features are specified in **Supplementary Tables 10-12**.

[d] Besides the ones without any TIL information, there were seven melanomas without the TIL type available.

[e] Precursor lesion, precursor type, microsatellites, regression, lymphovascular invasion, and perineural invasion were assumed absent if not listed as present in the pathology report. Melanomas without pathology reports available and melanomas with positive microsatellites were excluded from this study.

Supplementary Table 2. Features after one-hot encoding.

Demographics	Medical history	Tumor characteristics
1. Sex: Male	1. CCS	1. Type: Lentigo.maligna
2. Race: White	2. HPCM: Yes	2. Type: Melanoma.NOS
3. Ethnicity: Hispanic	3. HNMSC: Yes	3. Type: Nodular
4. Ethnicity: Non-Hispanic	4. HNMSC: No	4. Type: Superficial.spreading
5. Ethnicity: Unavailable	5. HNMSC: Unavailable	5. Site: Face
6. Median income	6. HSBN: Yes	6. Site: Lower.limb.and.hip
7. Insurance: Medicaid	7. HSBN: No	7. Site: Scalp.and.neck
8. Insurance: Medicare	8. HSBN: Unavailable	8. Site: Trunk
9. Insurance: Private	9. HOM: Yes	9. Site: Upper.limb.and.shoulder
10 Insurance: Self pay	10. HOM: No	10. Stage: 1A
11. Marital status: Divorced	11. HOM: Unavailable	11. Stage: 1B
12. Marital status: Married	12. HCAID: Yes	12. Stage: 2A
13. Marital status: Single	13: HCAID: No	13. Stage: 2B
14. Marital status: Widowed	14: HCAID: Unavailable	14. Stage: 2C
15. Marital status: Other	15: HSAID: Yes	15. Thickness
16. Marital status: Unavailable	16: HSAID: No	16. Anatomic level
17. Age at diagnosis	17: HSAID: Unavailable	17. Mitotic rate
		18. Ulceration: Absent
		19. Ulceration: Present
		20. Ulceration: Unavailable
		21. Laterality: Left
		22. Laterality: Midline
		23. Laterality: Right
		24. Total margins
		25. Margin check: Yes
		26. Margin check: No
		27. Margin check: Unknown
		28. RLNH: Not indicated
		29. RLNH: Negative
		30. RLNH: Age/Comorbidity
		31. RLNH: Unknown reason
		32. RLNH: Deferred
		33. TIL: Present
		34. TIL: Absent
		35. TIL: Unavailable
		36. TIL type: Absent
		37. TIL type: Brisk
		38. TIL type: Non-brisk
		39. TIL type: Unknown type
		40. TIL type: Unavailable
		41. Radial growth: Absent
		42. Radial growth: Present
		43. Radial growth: Unavailable
		44. Vertical growth: Absent
		45. Vertical growth: Present
		46. Vertical growth: Unavailable
		47. VGT: Absent
		48. VGT: Epithelioid
		49. VGT: Epithelioid.and.nevoid
		50. VGT: Epithelioid.and.small.cell
		51. VGT: Epithelioid.and.spindled
		52. VGT: Other

		53. VGT: Small.cell<br>54. VGT: Spindled<br>55. VGT: Unknown type<br>56. VGT: Unavailable<br>57. Precursor lesion: Present<br>58. Precursor type: Absent<br>59. Precursor type: Benign.nevus<br>60. Precursor type: Dermal.nevus<br>61. Precursor type: Dysplastic.nevus<br>62. Precursor type: Lentigo.maligna<br>63. Precursor type: Unknown type<br>64. Regression: Present<br>65. LVI: Present<br>66. Perineural invasion: Present

Supplementary Table 3. Characteristics of the entire study population.

Characteristics	Non-recurrence (N=1,410)	Recurrence (N=310)	P-value
**Institution**			
DFCI	453 (32.1%)	95 (30.6%)	0.660
MGB	957 (67.9%)	215 (69.4%)	
**Duration of follow-up (year)**			
Mean (SD)	8.4 (5.5)	6.7 (4.5)	<0.001
Median [IQR]	7.3 [3.8, 12.2]	5.8 [3.2, 9.3]	
**Recurrence type**			
Distant	Not applicable	151 (48.7%)	
Locoregional	Not applicable	159 (51.3%)	
**Time to recurrence (year)**			
Mean (SD)	Not applicable	2.9 (2.8)	
Median [IQR]	Not applicable	1.9 [0.9, 3.9]	
**Time to locoregional recurrence (year)**			
Mean (SD)	Not applicable	2.5 (2.6)	
Median [IQR]	Not applicable	1.5 [0.9, 3.1]	
**Time to distant recurrence (year)**			
Mean (SD)	Not applicable	3.4 (2.9)	
Median [IQR]	Not applicable	2.5 [1.3, 4.5]	
**Mortality status**			
Alive	1106 (78.4%)	158 (51.0%)	<0.001
Dead	304 (21.6%)	152 (49.0%)	
**Year of diagnosis**			
2000 - 2005	364 (25.8%)	59 (19.0%)	0.020
2006 - 2010	388 (27.5%)	88 (28.4%)	
2011 - 2015	514 (36.5%)	117 (37.7%)	
2016 - 2020	144 (10.2%)	46 (14.8%)	
**Age at diagnosis (year)**			
Mean (SD)	58.1 (15.7)	64.6 (14.9)	<0.001
Median [IQR]	60 [47, 70]	65 [55, 76]	
**Sex**			
Female	638 (45.2%)	112 (36.1%)	0.004
Male	772 (54.8%)	198 (63.9%)	
**Race**			
White	1399 (99.2%)	304 (98.1%)	0.122
Unavailable/Other	11 (0.8%)	6 (1.9%)	
**Ethnicity**			
Non-Hispanic	5 (0.4%)	0 (0%)	0.518
Hispanic	1387 (98.4%)	305 (98.4%)	
Unavailable	18 (1.3%)	5 (1.6%)	
**Median income (thousands of dollars)**			
Mean (SD)	105 (35.6)	102 (33.0)	0.175
Median [IQR]	101 [78.6, 126.7]	96.6 [79.0, 116.3]	
**Insurance Type**			
Medicaid	16 (1.1%)	10 (3.2%)	<0.001
Medicare	682 (48.4%)	172 (55.5%)	
Private	628 (44.5%)	73 (23.5%)	
Self-Pay	84 (6.0%)	55 (17.7%)	
**Marital status**			
Divorced	88 (6.2%)	22 (7.1%)	<0.001
Married	990 (70.2%)	206 (66.5%)	
Other	10 (0.7%)	0 (0%)	
Single	209 (14.8%)	36 (11.6%)	
Unavailable	33 (2.3%)	8 (2.6%)	
Widowed	80 (5.7%)	38 (12.3%)	
**History of cutaneous melanoma**			
No	954 (67.7%)	207 (66.8%)	0.815
Yes	456 (32.3%)	103 (33.2%)	
**Charlson comorbidity score**			
Mean (SD)	2.20 (2.13)	2.68 (2.19)	<0.001
Median [IQR]	2.0 [1.0, 3.0]	2.0 [1.0, 4.0]	
**History of non-melanoma skin cancer**			
Yes	816 (57.9%)	158 (51.0%)	<0.001
Unavailable	302 (21.4%)	111 (35.8%)	
No	292 (20.7%)	41 (13.2%)	
**History of situ or benign neoplasm of skin**			
No	1101 (78.1%)	197 (63.5%)	<0.001

Unavailable	302 (21.4%)	111 (35.8%)	
Yes	7 (0.5%)	2 (0.6%)	
**History of other malignancy**			
No	808 (57.3%)	139 (44.8%)	<0.001
Unavailable	302 (21.4%)	111 (35.8%)	
Yes	300 (21.3%)	60 (19.4%)	
**History of cutaneous autoimmune disease**			
No	1041 (73.8%)	187 (60.3%)	<0.001
Unavailable	302 (21.4%)	111 (35.8%)	
Yes	67 (4.8%)	12 (3.9%)	
**History of systemic autoimmune disease**			
No	968 (68.7%)	168 (54.2%)	<0.001
Unavailable	302 (21.4%)	111 (35.8%)	
Yes	140 (9.9%)	31 (10.0%)	
**Histology type**			
Lentigo maligna melanoma	107 (7.6%)	23 (7.4%)	<0.001
Melanoma, NOS	269 (19.1%)	67 (21.6%)	
Nodular melanoma	115 (8.2%)	85 (27.4%)	
Superficial spreading melanoma	919 (65.2%)	135 (43.5%)	
**Site**			
Skin of face	147 (10.4%)	51 (16.5%)	<0.001
Skin of lower limb and hip	268 (19.0%)	65 (21.0%)	
Skin of scalp and neck	84 (6.0%)	46 (14.8%)	
Skin of trunk	542 (38.4%)	89 (28.7%)	
Skin of upper limb and shoulder	369 (26.2%)	59 (19.0%)	
**AJCC Stage**			
1A	862 (61.1%)	49 (15.8%)	<0.001
1B	386 (27.4%)	84 (27.1%)	
2A	71 (5.0%)	58 (18.7%)	
2B	62 (4.4%)	63 (20.3%)	
2C	29 (2.1%)	56 (18.1%)	
**Breslow thickness**			
Mean (SD)	1.0 (1.4)	3.0 (4.2)	<0.001
Median [IQR]	0.6 [0.04, 1.1]	2.0 [1.1, 3.9]	
**Anatomic level**			
Mean (SD)	3.2 (0.9)	4.0 (0.8)	<0.001
Median [IQR]	3.0 [2.0, 4.0]	4.0 [4.00, 4.0]	
**Laterality**			
Left	669 (47.4%)	148 (47.7%)	0.626
Midline	123 (8.7%)	32 (10.3%)	
Right	0.989 (1.39)	3.01 (4.22)	<0.001
**Regional lymph node histology**			
Not indicated	826 (58.6%)	55 (17.7%)	<0.001
All nodes negative	519 (36.8%)	175 (56.5%)	
Not performed: unknown reason	30 (2.1%)	12 (3.9%)	
Not performed: due to age/comorbidity	30 (2.1%)	59 (19.0%)	
Not performed: deferred by patient	5 (0.4%)	9 (2.9%)	
**Ulceration**			
Absent	1305 (92.6%)	210 (67.7%)	<0.001
Present	101 (7.2%)	99 (31.9%)	
Unavailable	4 (0.3%)	1 (0.3%)	
**Mitotic rate (mitoses/mm^2)**			
Mean (SD)	1.8 (3.5)	7.6 (11.0)	<0.001
Median [IQR]	1.0 [0.0, 2.0]	4.0 [2.0, 10.0]	
**Total surgical margins (cm)**			
Mean (SD)	1.2 (0.5)	1.6 (0.5)	<0.001
Median [IQR]	1.0 [1.0, 1.5]	2.0 [1.0, 2.0]	
**Margin check**			
Yes	1128 (80.0%)	258 (83.2%)	0.337
No	78 (5.5%)	17 (5.5%)	
Unknown	204 (14.5%)	35 (11.3%)	
**Tumor infiltrating lymphocytes**			
Absent	299 (21.2%)	50 (16.1%)	<0.001
Present	736 (52.2%)	223 (71.9%)	
Unavailable	375 (26.6%)	37 (11.9%)	
**Tumor infiltrating lymphocyte type**			
Absent	299 (21.2%)	50 (16.1%)	<0.001
Brisk	105 (7.4%)	15 (4.8%)	
Non-Brisk	624 (44.3%)	208 (67.1%)	
Unavailable	375 (26.6%)	37 (11.9%)	

Unknown type	7 (0.5%)	0 (0%)	
**Radial growth phase**			
Absent	118 (8.4%)	89 (28.7%)	<0.001
Present	1181 (83.8%)	192 (61.9%)	
Unavailable	111 (7.9%)	29 (9.4%)	
**Vertical growth phase**			
Absent	284 (20.1%)	15 (4.8%)	<0.001
Present	976 (69.2%)	275 (88.7%)	
Unavailable	150 (10.6%)	20 (6.5%)	
**Vertical growth type**			
Absent	284 (20.1%)	15 (4.8%)	<0.001
Epithelioid	628 (44.5%)	162 (52.3%)	
Epithelioid and nevoid	26 (1.8%)	1 (0.3%)	
Epithelioid and small cell	52 (3.7%)	9 (2.9%)	
Epithelioid and spindled	103 (7.3%)	62 (20.0%)	
Other	22 (1.6%)	3 (1.0%)	
Small Cell	14 (1.0%)	1 (0.3%)	
Spindled	39 (2.8%)	17 (5.5%)	
Unavailable	150 (10.6%)	20 (6.5%)	
Unknown type	92 (6.5%)	20 (6.5%)	
**Precursor lesion**			
Absent	924 (65.5%)	257 (82.9%)	<0.001
Present	486 (34.5%)	53 (17.1%)	
**Precursor type**			
Absent	924 (65.5%)	257 (82.9%)	<0.001
Benign Nevus	226 (16.0%)	24 (7.7%)	
Dermal Nevus	3 (0.2%)	0 (0%)	
Dysplastic Nevus	220 (15.6%)	23 (7.4%)	
Lentigo Maligna	22 (1.6%)	6 (1.9%)	
Unknown type	15 (1.1%)	0 (0%)	
**Microsatellites** [a]			
Absent	1410 (100%)	310 (100%)	<0.001
**Regression**			
Absent	1187 (84.2%)	287 (92.6%)	<0.001
Present	223 (15.8%)	23 (7.4%)	
**Lymphovascular invasion**			
Absent	1391 (98.7%)	292 (94.2%)	<0.001
Present	19 (1.3%)	18 (5.8%)	
**Perineural invasion**			
Absent	1391 (98.7%)	299 (96.5%)	0.0147
Present	19 (1.3%)	11 (3.5%)	

[a] Samples with positive microsatellites were excluded in this study.

Supplementary Table 4. Characteristics of the MGB and DFCI cohorts for classification tasks.

Characteristics	MGB cohort			DFCI cohort		
	Non-recurrence (N=666)	Recurrence (N=215)	P-value	Non-recurrence (N=340)	Recurrence (N=95)	P-value
**Duration of follow-up (year)**						
Mean (SD)	12.7 (4.5)	7.1 (5.0)	<0.001	7.3 (1.4)	5.9 (2.9)	<0.001
Median [IQR]	12.6 [8.9, 16.0]	5.8 [3.1, 10.7]		7.1 [6.3, 8.1]	5.8 [3.4, 8.3]	
**Recurrence type**						
Distant	Not applicable	98 (45.6%)		Not applicable	53 (55.8%)	
Locoregional	Not applicable	117 (54.4%)		Not applicable	42 (44.2%)	
**Time to recurrence (year)**						
Mean (SD)	Not applicable	3.1 (3.1)		Not applicable	2.6 (2.0)	
Median [IQR]	Not applicable	1.9 [0.9, 4.2]		Not applicable	1.8 [1.1, 3.5]	
**Time to locoregional recurrence**						
Mean (SD)	Not applicable	2.7 (2.9)		Not applicable	1.8 (1.6)	
Median [IQR]	Not applicable	1.6 [0.8, 3.7]		Not applicable	1.4 [0.7, 2.3]	
**Time to distant recurrence**						
Mean (SD)	Not applicable	3.5 (3.3)		Not applicable	3.1 (2.1)	
Median [IQR]	Not applicable	2.3 [1.1, 4.4]		Not applicable	2.7 [1.6, 4.5]	
**Mortality status**						
Alive	461 (69.2%)	100 (46.5%)	<0.001	302 (88.8%)	58 (61.1%)	<0.001
Dead	205 (30.8%)	115 (53.5%)		38 (11.2%)	37 (38.9%)	
**Year of diagnosis**						
2000 - 2005	320 (48.0%)	59 (27.4%)	<0.001	0 (0%)	0 (0%)	<0.001
2006 - 2010	288 (43.2%)	77 (35.8%)		18 (5.3%)	11 (11.6%)	
2011 - 2015	58 (8.7%)	59 (27.4%)		317 (93.2%)	58 (61.1%)	
2016 - 2020	0 (0%)	20 (9.3%)		5 (1.5%)	26 (27.4%)	
**Age at diagnosis**						
Mean (SD)	57.6 (16.0)	64.9 (15.2)	<0.001	59.5 (13.9)	63.9 (14.4)	0.009
Median [IQR]	60.0 [46.0, 69.0]	65.0 [55.5, 77.0]		61.0 [51.0, 69.2]	64.0 [55.5, 73.0]	
**Gender**						
Female	270 (40.5%)	81 (37.7%)	0.505	174 (51.2%)	31 (32.6%)	0.002
Male	396 (59.5%)	134 (62.3%)		166 (48.8%)	64 (67.4%)	
**Race**						
Other/Unknown	2 (0.3%)	3 (1.4%)	0.181	3 (0.9%)	3 (3.2%)	0.237
White	664 (99.7%)	212 (98.6%)		337 (99.1%)	92 (96.8%)	
**Ethnicity**						
Hispanic	1 (0.2%)	0 (0%)	0.417	4 (1.2%)	0 (0%)	0.540
Non-Hispanic	663 (99.5%)	213 (99.1%)		323 (95.0%)	92 (96.8%)	
Unknown	2 (0.3%)	2 (0.9%)		13 (3.8%)	3 (3.2%)	
**Median income (thousands of dollars)**						
Mean (SD)	105 (36.)	102 (33)	0.317	102 (34)	100 (33)	0.670
Median [IQR]	102 [77, 128]	98 [81, 102]		97 [78, 123]	93 [77, 115]	
**Insurance type**						
Medicaid	12 (1.8%)	6 (2.8%)	<0.001	4 (1.2%)	4 (4.2%)	0.009
Medicare	342 (51.4%)	117 (54.4%)		175 (51.5%)	55 (57.9%)	
Private	250 (37.5%)	46 (21.4%)		146 (42.9%)	27 (28.4%)	
Self-Pay	62 (9.3%)	46 (21.4%)		15 (4.4%)	9 (9.5%)	
**Marital status**						
Divorced	39 (5.9%)	15 (7.0%)	0.095	27 (7.9%)	7 (7.4%)	0.584
Married	451 (67.7%)	138 (64.2%)		249 (73.2%)	68 (71.6%)	
Single	102 (15.3%)	26 (12.1%)		42 (12.4%)	10 (10.5%)	
Unavailable	23 (3.5%)	7 (3.3%)		5 (1.5%)	1 (1.1%)	
Widowed	51 (7.7%)	29 (13.5%)		17 (5.0%)	9 (9.5%)	
**History of cutaneous melanoma**						
No	464 (69.7%)	140 (65.1%)	0.244	223 (65.6%)	67 (70.5%)	0.436
Yes	202 (30.3%)	75 (34.9%)		117 (34.4%)	28 (29.5%)	
**Charlson comorbidity score**						
Mean (SD)	2.1 (2.1)	2.8 (2.2)	<0.001	2.3 (2.1)	2.5 (2.1)	0.378
Median [IQR]	2.0 [0, 3.0]	3.0 [1.0, 4.0]		2.0 [1.0, 3.0]	2.0 [1.0, 3.0]	
**History of non-melanoma skin cancer**						
Yes	369 (55.4%)	110 (51.2%)	<0.001	199 (58.5%)	48 (50.5%)	0.070
Unavailable	137 (20.6%)	76 (35.3%)		85 (25.0%)	35 (36.8%)	
No	160 (24.0%)	29 (13.5%)		56 (16.5%)	12 (12.6%)	
**History of situ or benign neoplasm of skin**						

No	529 (79.4%)	139 (64.7%)	<0.001	255 (75.0%)	58 (61.1%)	0.002
Unavailable	137 (20.6%)	76 (35.3%)		85 (25.0%)	35 (36.8%)	
Yes	0 (0%)	0 (0%)		0 (0%)	2 (2.1%)	
**History of other malignancy**						
No	408 (61.3%)	95 (44.2%)	<0.001	182 (53.5%)	44 (46.3%)	0.071
Unavailable	137 (20.6%)	76 (35.3%)		85 (25.0%)	35 (36.8%)	
Yes	121 (18.2%)	44 (20.5%)		73 (21.5%)	16 (16.8%)	
**History of cutaneous autoimmune disease**						
No	495 (74.3%)	131 (60.9%)	<0.001	242 (71.2%)	56 (58.9%)	0.066
Unavailable	137 (20.6%)	76 (35.3%)		85 (25.0%)	35 (36.8%)	
Yes	34 (5.1%)	8 (3.7%)		13 (3.8%)	4 (4.2%)	
**History of systemic autoimmune disease**						
No	467 (70.1%)	118 (54.9%)	<0.001	223 (65.6%)	50 (52.6%)	0.054
Unavailable	137 (20.6%)	76 (35.3%)		85 (25.0%)	35 (36.8%)	
Yes	62 (9.3%)	21 (9.8%)		32 (9.4%)	10 (10.5%)	
**Histology type**						
Lentigo maligna melanoma	76 (11.4%)	20 (9.3%)	<0.001	13 (3.8%)	3 (3.2%)	<0.001
Melanoma, NOS	61 (9.2%)	27 (12.6%)		137 (40.3%)	40 (42.1%)	
Nodular melanoma	55 (8.3%)	65 (30.2%)		22 (6.5%)	20 (21.1%)	
Superficial spreading melanoma	474 (71.2%)	103 (47.9%)		168 (49.4%)	32 (33.7%)	
**Site**						
Skin of face	79 (11.9%)	33 (15.3%)	<0.001	39 (11.5%)	18 (18.9%)	<0.001
Skin of lower limb and hip	92 (13.8%)	47 (21.9%)		75 (22.1%)	18 (18.9%)	
Skin of scalp and neck	38 (5.7%)	26 (12.1%)		24 (7.1%)	20 (21.1%)	
Skin of trunk	278 (41.7%)	66 (30.7%)		110 (32.4%)	23 (24.2%)	
Skin of upper limb and shoulder	179 (26.9%)	43 (20.0%)		92 (27.1%)	16 (16.8%)	
**AJCC Stage**						
1A	424 (63.7%)	38 (17.7%)	<0.001	204 (60.0%)	11 (11.6%)	<0.001
1B	181 (27.2%)	51 (23.7%)		91 (26.8%)	33 (34.7%)	
2A	25 (3.8%)	44 (20.5%)		20 (5.9%)	14 (14.7%)	
2B	26 (3.9%)	40 (18.6%)		19 (5.6%)	23 (24.2%)	
2C	10 (1.5%)	42 (19.5%)		6 (1.8%)	14 (14.7%)	
**Breslow thickness**						
Mean (SD)	0.9 (0.8)	3.0 (4.6)	<0.001	1.0 (1.3)	3.0 (3.2)	<0.001
Median [IQR]	0.6 [0.4, 1.1]	2.1 [1.0, 4.0]		0.6 [0.4, 1.2]	2.0 [1.1, 3.8]	
**Anatomic level**						
Mean (SD)	3.1 (0.9)	3.9 (0.7)	<0.001	3.3 (1.0)	4.3 (1.0)	<0.001
Median [IQR]	3.0 [2.0, 4.0]	4.0 [4.0, 4.0]		3.0 [2.8, 4.0]	4.0 [4.0, 4.0]	
**Laterality**						
Left	311 (46.7%)	110 (51.2%)	0.336	156 (45.9%)	38 (40.0%)	0.513
Midline	63 (9.5%)	23 (10.7%)		24 (7.1%)	9 (9.5%)	
Right	292 (43.8%)	82 (38.1%)		160 (47.1%)	48 (50.5%)	
**Regional lymph node histology**						
Not indicated	446 (67.0%)	42 (19.5%)	<0.001	156 (45.9%)	13 (13.7%)	<0.001
All nodes negative	167 (25.1%)	110 (51.2%)		172 (50.6%)	65 (68.4%)	
Not performed: unknown reason	25 (3.8%)	10 (4.7%)		5 (1.5%)	2 (2.1%)	
Not performed: age/comorbidity	25 (3.8%)	46 (21.4%)		5 (1.5%)	13 (13.7%)	
Not performed: deferred by patient	3 (0.5%)	7 (3.3%)		2 (0.6%)	2 (2.1%)	
**Ulceration**						
Absent	620 (93.1%)	144 (67.0%)	<0.001	315 (92.6%)	66 (69.5%)	<0.001
Present	45 (6.8%)	71 (33.0%)		22 (6.5%)	28 (29.5%)	
Unavailable	1 (0.2%)	0 (0%)		3 (0.9%)	1 (1.1%)	
**Mitotic rate (mitoses/mm^2)**						
Mean (SD)	1.6 (2.9)	7.8 (12.3)	<0.001	1.8 (3.3)	7.4 (7.5)	<0.001
Median [IQR]	1.0 [0.0, 1.0]	4.0 [2.0, 10.0]		1.0 [0.0, 2.0]	4.0 [2.0, 11.0]	
**Total margins**						
Mean (SD)	1.2 (0.5)	1.6 (0.5)	<0.001	1.3 (0.5)	1.5 (0.5)	<0.001
Median [IQR]	1.0 [1.0, 1.5]	2.0 [1.0, 2.0]		1.0 [1.0, 1.5]	1.5 [1.0, 2.0]	
**Margin check**						
Yes	500 (75.1%)	181 (84.2%)	0.00495	287 (84.4%)	77 (81.1%)	0.698
No	37 (5.6%)	13 (6.0%)		10 (2.9%)	4 (4.2%)	
Unknown	129 (19.4%)	21 (9.8%)		43 (12.6%)	14 (14.7%)	
**Tumor infiltrating lymphocytes**						
Absent	139 (20.9%)	39 (18.1%)	<0.001	34 (10.0%)	11 (11.6%)	0.191
Present	285 (42.8%)	158 (73.5%)		206 (60.6%)	65 (68.4%)	
Unavailable	242 (36.3%)	18 (8.4%)		100 (29.4%)	19 (20.0%)	
**Tumor infiltrating lymphocyte type**						
Absent	139 (20.9%)	39 (18.1%)	<0.001	34 (10.0%)	11 (11.6%)	0.048

Brisk	34 (5.1%)	11 (5.1%)		35 (10.3%)	4 (4.2%)	
Non-Brisk	248 (37.2%)	147 (68.4%)		168 (49.4%)	61 (64.2%)	
Unavailable	242 (36.3%)	18 (8.4%)		100 (29.4%)	19 (20.0%)	
Unknown type	3 (0.5%)	0 (0%)		3 (0.9%)	0 (0%)	
**Radial growth phase**						
Absent	68 (10.2%)	70 (32.6%)	<0.001	14 (4.1%)	19 (20.0%)	<0.001
Present	579 (86.9%)	137 (63.7%)		281 (82.6%)	55 (57.9%)	
Unavailable	19 (2.9%)	8 (3.7%)		45 (13.2%)	21 (22.1%)	
**Vertical growth phase**						
Absent	213 (32.0%)	14 (6.5%)	<0.001	9 (2.6%)	1 (1.1%)	0.187
Present	429 (64.4%)	198 (92.1%)		245 (72.1%)	77 (81.1%)	
Unavailable	24 (3.6%)	3 (1.4%)		86 (25.3%)	17 (17.9%)	
**Vertical growth type**						
Absent	213 (32.0%)	14 (6.5%)	<0.001	9 (2.6%)	1 (1.1%)	0.004
Epithelioid	273 (41.0%)	118 (54.9%)		159 (46.8%)	44 (46.3%)	
Epithelioid and nevoid	3 (0.5%)	0 (0%)		17 (5.0%)	1 (1.1%)	
Epithelioid and small cell	24 (3.6%)	7 (3.3%)		12 (3.5%)	2 (2.1%)	
Epithelioid and spindled	46 (6.9%)	42 (19.5%)		29 (8.5%)	20 (21.1%)	
Other	5 (0.8%)	2 (0.9%)		8 (2.4%)	1 (1.1%)	
Small Cell	9 (1.4%)	1 (0.5%)		3 (0.9%)	0 (0%)	
Spindled	25 (3.8%)	17 (7.9%)		5 (1.5%)	0 (0%)	
Unavailable	24 (3.6%)	3 (1.4%)		86 (25.3%)	17 (17.9%)	
Unknown type	44 (6.6%)	11 (5.1%)		12 (3.5%)	9 (9.5%)	
**Precursor lesion**						
Absent	403 (60.5%)	172 (80.0%)	<0.001	254 (74.7%)	85 (89.5%)	0.003
Present	263 (39.5%)	43 (20.0%)		86 (25.3%)	10 (10.5%)	
**Precursor type**						
Absent	403 (60.5%)	172 (80.0%)	<0.001	254 (74.7%)	85 (89.5%)	0.009
Benign Nevus	103 (15.5%)	20 (9.3%)		50 (14.7%)	4 (4.2%)	
Dermal Nevus	1 (0.2%)	0 (0%)		2 (0.6%)	0 (0%)	
Dysplastic Nevus	136 (20.4%)	18 (8.4%)		28 (8.2%)	5 (5.3%)	
Lentigo Maligna	20 (3.0%)	5 (2.3%)		6 (1.8%)	0 (0%)	
Unknown type	3 (0.5%)	0 (0%)		0 (0%)	1 (1.1%)	
**Regression**						
Absent	605 (90.8%)	202 (94.0%)	0.197	249 (73.2%)	85 (89.5%)	0.001
Present	61 (9.2%)	13 (6.0%)		91 (26.8%)	10 (10.5%)	
**Lymphovascular invasion**						
Absent	662 (99.4%)	202 (94.0%)	<0.001	335 (98.5%)	90 (94.7%)	0.073
Present	4 (0.6%)	13 (6.0%)		5 (1.5%)	5 (5.3%)	
**Perineural invasion**						
Absent	658 (98.8%)	207 (96.3%)	0.0347	337 (99.1%)	92 (96.8%)	0.237
Present	8 (1.2%)	8 (3.7%)		3 (0.9%)	3 (3.2%)	

Supplementary Table 5. Characteristics of the MGB and DFCI cohorts for time-to-event prediction tasks.

Characteristics	MGB cohort			DFCI cohort		
	Non-recurrence (N=645)	Recurrence (N=215)	P-value	Non-recurrence (N=285)	Recurrence (N=95)	P-value
**Duration of follow-up (year)**						
Mean (SD)	7.0 (5.5)	7.1 (5.0)	0.763	5.7 (2.6)	5.9 (2.9)	0.400
Median [IQR]	6.1 [1.6, 11.5]	5.8 [3.1, 10.7]		5.8 [3.4, 7.8]	5.8 [3.4, 8.3]	
**Year of diagnosis**						
2000 - 2005	180 (27.9%)	59 (27.4%)	0.585	0 (0%)	0 (0%)	0.225
2006 - 2010	249 (38.6%)	77 (35.8%)		18 (6.3%)	11 (11.6%)	
2011 - 2015	148 (22.9%)	59 (27.4%)		191 (67.0%)	58 (61.1%)	
2016 - 2020	68 (10.5%)	20 (9.3%)		76 (26.7%)	26 (27.4%)	
**Age at diagnosis**						
Mean (SD)	58.4 (16.7)	64.9 (15.2)	<0.001	60.0 (14.1)	63.9 (14.4)	0.024
Median [IQR]	61.0 [47.0, 71.0]	65.0 [55.5, 77.0]		62.0 [52.0, 70.0]	64.0 [55.5, 73.0]	
**Gender**						
Female	286 (44.3%)	81 (37.7%)	0.103	141 (49.5%)	31 (32.6%)	0.006
Male	359 (55.7%)	134 (62.3%)		144 (50.5%)	64 (67.4%)	
**Race**						
Other/Unknown	5 (0.8%)	3 (1.4%)	0.682	4 (1.4%)	3 (3.2%)	0.509
White	640 (99.2%)	212 (98.6%)		281 (98.6%)	92 (96.8%)	
**Ethnicity**						
Hispanic	0 (0%)	0 (0%)	0.563	2 (0.7%)	0 (0%)	0.705
Non-Hispanic	643 (99.7%)	213 (99.1%)		275 (96.5%)	92 (96.8%)	
Unknown	2 (0.3%)	2 (0.9%)		8 (2.8%)	3 (3.2%)	
**Median income (thousands of dollars)**						
Mean (SD)	103 (35)	102 (33)	0.893	104 (34)	100 (33)	0.373
Median [IQR]	99 [78, 123]	98 [81, 102]		101 [80, 127]	93 [77, 115]	
**Insurance type**						
Medicaid	7 (1.1%)	6 (2.8%)	<0.001	2 (0.7%)	4 (4.2%)	<0.001
Medicare	287 (44.5%)	117 (54.4%)		151 (53.0%)	55 (57.9%)	
Private	296 (45.9%)	46 (21.4%)		126 (44.2%)	27 (28.4%)	
Self-Pay	55 (8.5%)	46 (21.4%)		6 (2.1%)	9 (9.5%)	
**Marital status**						
Divorced	34 (5.3%)	15 (7.0%)	0.010	19 (6.7%)	7 (7.4%)	0.496
Married	444 (68.8%)	138 (64.2%)		213 (74.7%)	68 (71.6%)	
Other	9 (1.4%)	0 (0%)		1 (0.4%)	0 (0%)	
Single	98 (15.2%)	26 (12.1%)		36 (12.6%)	10 (10.5%)	
Unavailable	18 (2.8%)	7 (3.3%)		4 (1.4%)	1 (1.1%)	
Widowed	42 (6.5%)	29 (13.5%)		12 (4.2%)	9 (9.5%)	
**History of cutaneous melanoma**						
No	450 (69.8%)	140 (65.1%)	0.244	176 (61.8%)	67 (70.5%)	0.156
Yes	195 (30.2%)	75 (34.9%)		109 (38.2%)	28 (29.5%)	
**Charlson comorbidity score**						
Mean (SD)	2.3 (2.2)	2.8 (2.2)	0.006	2.4 (2.1)	2.5 (2.1)	0.798
Median [IQR]	2.0 [0, 3.0]	3.0 [1.0, 4.0]		2.00 [1.0, 3.0]	2.0 [1.0, 3.0]	
**History of non-melanoma skin cancer**						
Yes	360 (55.8%)	110 (51.2%)	<0.001	170 (59.6%)	48 (50.5%)	<0.001
Unavailable	150 (23.3%)	76 (35.3%)		53 (18.6%)	35 (36.8%)	
No	135 (20.9%)	29 (13.5%)		62 (21.8%)	12 (12.6%)	
**History of situ or benign neoplasm of skin**						
No	492 (76.3%)	139 (64.7%)	0.002	228 (80.0%)	58 (61.1%)	<0.001
Unavailable	150 (23.3%)	76 (35.3%)		53 (18.6%)	35 (36.8%)	
Yes	3 (0.5%)	0 (0%)		4 (1.4%)	2 (2.1%)	
**History of other malignancy**						
No	365 (56.6%)	95 (44.2%)	0.001	156 (54.7%)	44 (46.3%)	<0.001
Unavailable	150 (23.3%)	76 (35.3%)		53 (18.6%)	35 (36.8%)	
Yes	130 (20.2%)	44 (20.5%)		76 (26.7%)	16 (16.8%)	
**History of cutaneous autoimmune disease**						
No	461 (71.5%)	131 (60.9%)	0.002	216 (75.8%)	56 (58.9%)	0.001

Unavailable	150 (23.3%)	76 (35.3%)		53 (18.6%)	35 (36.8%)	
Yes	34 (5.3%)	8 (3.7%)		16 (5.6%)	4 (4.2%)	
**History of systemic autoimmune disease**						
No	432 (67.0%)	118 (54.9%)	0.002	194 (68.1%)	50 (52.6%)	0.001
Unavailable	150 (23.3%)	76 (35.3%)		53 (18.6%)	35 (36.8%)	
Yes	63 (9.8%)	21 (9.8%)		38 (13.3%)	10 (10.5%)	
**Histology type**						
Lentigo maligna melanoma	55 (8.5%)	20 (9.3%)	<0.001	16 (5.6%)	3 (3.2%)	<0.001
Melanoma, NOS	68 (10.5%)	27 (12.6%)		110 (38.6%)	40 (42.1%)	
Nodular melanoma	59 (9.1%)	65 (30.2%)		18 (6.3%)	20 (21.1%)	
Superficial spreading melanoma	463 (71.8%)	103 (47.9%)		141 (49.5%)	32 (33.7%)	
**Site**						
Skin of face	67 (10.4%)	33 (15.3%)	<0.001	36 (12.6%)	18 (18.9%)	<0.001
Skin of lower limb and hip	132 (20.5%)	47 (21.9%)		57 (20.0%)	18 (18.9%)	
Skin of scalp and neck	33 (5.1%)	26 (12.1%)		20 (7.0%)	20 (21.1%)	
Skin of trunk	241 (37.4%)	66 (30.7%)		96 (33.7%)	23 (24.2%)	
Skin of upper limb and shoulder	172 (26.7%)	43 (20.0%)		76 (26.7%)	16 (16.8%)	
**AJCC Stage**						
1A	383 (59.4%)	38 (17.7%)	<0.001	180 (63.2%)	11 (11.6%)	<0.001
1B	185 (28.7%)	51 (23.7%)		66 (23.2%)	33 (34.7%)	
2A	36 (5.6%)	44 (20.5%)		20 (7.0%)	14 (14.7%)	
2B	27 (4.2%)	40 (18.6%)		11 (3.9%)	23 (24.2%)	
2C	14 (2.2%)	42 (19.5%)		8 (2.8%)	14 (14.7%)	
**Breslow thickness**						
Mean (SD)	1.0 (1.6)	3.0 (4.6)	<0.001	1.1 (1.5)	3.0 (3.2)	<0.001
Median [IQR]	0.6 [0.4, 1.2]	2.1 [1.0, 4.0]		0.6 [0.4, 1.1]	2.0 [1.1, 3.8]	
**Anatomic level**						
Mean (SD)	3.2 (0.9)	3.9 (0.7)	<0.001	3.3 (0.9)	4.3 (1.0)	<0.001
Median [IQR]	3.0 [2.0, 4.0]	4.0 [4.0, 4.0]		3.0 [2.0, 4.0]	4.0[4.0, 4.0]	
**Laterality**						
Left	311 (48.2%)	110 (51.2%)	0.510	149 (52.3%)	38 (40.0%)	0.095
Midline	60 (9.3%)	23 (10.7%)		17 (6.0%)	9 (9.5%)	
Right	274 (42.5%)	82 (38.1%)		119 (41.8%)	48 (50.5%)	
**Regional lymph node histology**						
Not indicated	392 (60.8%)	42 (19.5%)	<0.001	141 (49.5%)	13 (13.7%)	<0.001
All nodes negative	221 (34.3%)	110 (51.2%)		141 (49.5%)	65 (68.4%)	
Not performed: unknown reason	13 (2.0%)	10 (4.7%)		1 (0.4%)	2 (2.1%)	
Not performed: age/comorbidity	18 (2.8%)	46 (21.4%)		1 (0.4%)	13 (13.7%)	
Not performed: deferred by patient	1 (0.2%)	7 (3.3%)		1 (0.4%)	2 (2.1%)	
**Ulceration**						
Absent	600 (93.0%)	144 (67.0%)	<0.001	262 (91.9%)	66 (69.5%)	<0.001
Present	45 (7.0%)	71 (33.0%)		23 (8.1%)	28 (29.5%)	
Unavailable	0 (0%)	0 (0%)		0 (0%)	1 (1.1%)	
**Mitotic rate (mitoses/mm^2)**						
Mean (SD)	2.0 (4.0)	7.8 (12.3)	<0.001	1.7 (3.3)	7.4 (7.5)	<0.001
Median [IQR]	1.0 [0.0, 2.0]	4.0 [2.0, 10.0]		1.0 [0.0, 2.0]	4.0 [2.0, 11.0]	
**Total margins**						
Mean (SD)	1.2 (0.5)	1.6 (0.5)	<0.001	1.3 (0.5)	1.5 (0.5)	<0.001
Median [IQR]	1.0 [1.0, 1.5]	2.0 [1.0, 2.0]		1.0 [1.0, 1.5]	1.5 [1.0, 2.0]	
**Margin check**						
Yes	516 (80.0%)	181 (84.2%)	0.301	247 (86.7%)	77 (81.1%)	0.159
No	40 (6.2%)	13 (6.0%)		15 (5.3%)	4 (4.2%)	
Unknown	89 (13.8%)	21 (9.8%)		23 (8.1%)	14 (14.7%)	
**Tumor infiltrating lymphocytes**						
Absent	166 (25.7%)	39 (18.1%)	<0.001	52 (18.2%)	11 (11.6%)	0.281
Present	323 (50.1%)	158 (73.5%)		174 (61.1%)	65 (68.4%)	
Unavailable	156 (24.2%)	18 (8.4%)		59 (20.7%)	19 (20.0%)	
**Tumor infiltrating lymphocyte type**						
Absent	166 (25.7%)	39 (18.1%)	<0.001	52 (18.2%)	11 (11.6%)	0.037
Brisk	35 (5.4%)	11 (5.1%)		34 (11.9%)	4 (4.2%)	
Non-Brisk	285 (44.2%)	147 (68.4%)		138 (48.4%)	61 (64.2%)	
Unavailable	156 (24.2%)	18 (8.4%)		59 (20.7%)	19 (20.0%)	
Unknown type	3 (0.5%)	0 (0%)		2 (0.7%)	0 (0%)	
**Radial growth phase**						
Absent	71 (11.0%)	70 (32.6%)	<0.001	8 (2.8%)	19 (20.0%)	<0.001
Present	538 (83.4%)	137 (63.7%)		227 (79.6%)	55 (57.9%)	
Unavailable	36 (5.6%)	8 (3.7%)		50 (17.5%)	21 (22.1%)	
**Vertical growth phase**						
Absent	166 (25.7%)	14 (6.5%)	<0.001	8 (2.8%)	1 (1.1%)	0.12

Present	452 (70.1%)	198 (92.1%)		201 (70.5%)	77 (81.1%)	
Unavailable	27 (4.2%)	3 (1.4%)		76 (26.7%)	17 (17.9%)	
**Vertical growth type**						
Absent	166 (25.7%)	14 (6.5%)	<0.001	8 (2.8%)	1 (1.1%)	0.072
Epithelioid	301 (46.7%)	118 (54.9%)		118 (41.4%)	44 (46.3%)	
Epithelioid and nevoid	5 (0.8%)	0 (0%)		12 (4.2%)	1 (1.1%)	
Epithelioid and small cell	26 (4.0%)	7 (3.3%)		7 (2.5%)	2 (2.1%)	
Epithelioid and spindled	40 (6.2%)	42 (19.5%)		27 (9.5%)	20 (21.1%)	
Other	7 (1.1%)	2 (0.9%)		7 (2.5%)	1 (1.1%)	
Small Cell	8 (1.2%)	1 (0.5%)		2 (0.7%)	0 (0%)	
Spindled	23 (3.6%)	17 (7.9%)		3 (1.1%)	0 (0%)	
Unavailable	27 (4.2%)	3 (1.4%)		76 (26.7%)	17 (17.9%)	
Unknown type	42 (6.5%)	11 (5.1%)		25 (8.8%)	9 (9.5%)	
**Precursor lesion**						
Absent	418 (64.8%)	172 (80.0%)	<0.001	204 (71.6%)	85 (89.5%)	<0.001
Present	227 (35.2%)	43 (20.0%)		81 (28.4%)	10 (10.5%)	
**Precursor type**						
Absent	418 (64.8%)	172 (80.0%)	<0.001	204 (71.6%)	85 (89.5%)	0.003
Benign Nevus	96 (14.9%)	20 (9.3%)		50 (17.5%)	4 (4.2%)	
Dermal Nevus	0 (0%)	0 (0%)		1 (0.4%)	0 (0%)	
Dysplastic Nevus	112 (17.4%)	18 (8.4%)		23 (8.1%)	5 (5.3%)	
Lentigo Maligna	15 (2.3%)	5 (2.3%)		7 (2.5%)	0 (0%)	
Unknown type	4 (0.6%)	0 (0%)		0 (0%)	1 (1.1%)	
**Regression**						
Absent	574 (89.0%)	202 (94.0%)	0.047	216 (75.8%)	85 (89.5%)	0.007
Present	71 (11.0%)	13 (6.0%)		69 (24.2%)	10 (10.5%)	
**Lymphovascular invasion**						
Absent	637 (98.8%)	202 (94.0%)	<0.001	282 (98.9%)	90 (94.7%)	0.039
Present	8 (1.2%)	13 (6.0%)		3 (1.1%)	5 (5.3%)	
**Perineural invasion**						
Absent	631 (97.8%)	207 (96.3%)	0.318	283 (99.3%)	92 (96.8%)	0.194
Present	14 (2.2%)	8 (3.7%)		2 (0.7%)	3 (3.2%)	

Supplementary Table 6. Model performances for recurrence classification.

		AUC	PPV	Sensitivity	Specificity	Accuracy
GB	Internal	**0.845 CI[b]:0.840-0.850**	**0.803 CI:0.797-0.810**	0.76 CI:0.754-0.767	0.784 CI:0.776-0.792	**0.772 CI:0.768-0.777**
	External	**0.812 CI:0.804-0.819**	**0.785 CI:0.774-0.795**	0.687 CI:0.678-0.696	0.794 CI:0.784-0.804	**0.741 CI:0.735-0.746**
	P-value[a]	<0.001	0.021	<0.001	0.305	<0.001
LR	Internal	0.828 CI:0.823-0.834	0.760 CI:0.754-0.766	0.758 CI:0.752-0.764	0.757 CI:0.749-0.765	0.758 CI:0.753-0.762
	External	0.784 CI:0.779-0.788	715 CI:0.708-0.723	0.664 CI:0.658-0.67	0.735 CI:0.727-0.744	0.700 CI:0.694-0.707
	P-value[a]	<0.001	<0.001	<0.001	0.020	<0.001
MLP	Internal	0.830 CI:0.824-0.836	0.759 CI:0.752-0.766	0.758 CI:0.753-0.764	0.755 CI:0.746-0.764	0.757 CI:0.751-0.762
	External	0.786 CI:0.779-0.792	0.711 CI:0.702-0.72	0.711 CI:0.702-0.72	0.711 CI:0.699-0.724	0.708 CI:0.701-0.715
	P-value[a]	<0.001	<0.001	<0.001	<0.001	<0.001
RF	Internal	0.842 CI:0.837-0.847	0.778 CI:0.771-0.785	0.756 CI:0.75-0.762	0.78 CI:0.771-0.788	0.768 CI:0.763-0.773
	External	0.791 CI:0.783-0.798	0.737 CI:0.727-0.748	0.709 CI:0.701-0.717	0.745 CI:0.73-0.76	0.727 CI:0.72-0.734
	P-value[a]	<0.001	0.422	<0.001	0.001	<0.001
SVM	Internal	0.811 CI:0.805-0.817	0.754 CI:0.747-0.76	0.736 CI:0.73-0.742	0.755 CI:0.746-0.764	0.749 CI:0.744-0.755
	External	0.767 CI:0.762-0.773	0.717 CI:0.708-0.725	0.701 CI:0.692-0.71	0.721 CI:0.709-0.732	0.716 CI:0.709-0.724
	P-value[a]	<0.001	<0.001	<0.001	0.001	<0.001

[a] P-value: comparison between the internal validation and external validation.

[b] CI: 95% Confidence Interval. The best performances were highlighted.

Supplementary Figure 2. ROC curves with 50 repeats for recurrence classification on the original cohorts.

a. ROC curves in internal and external validations using RF.

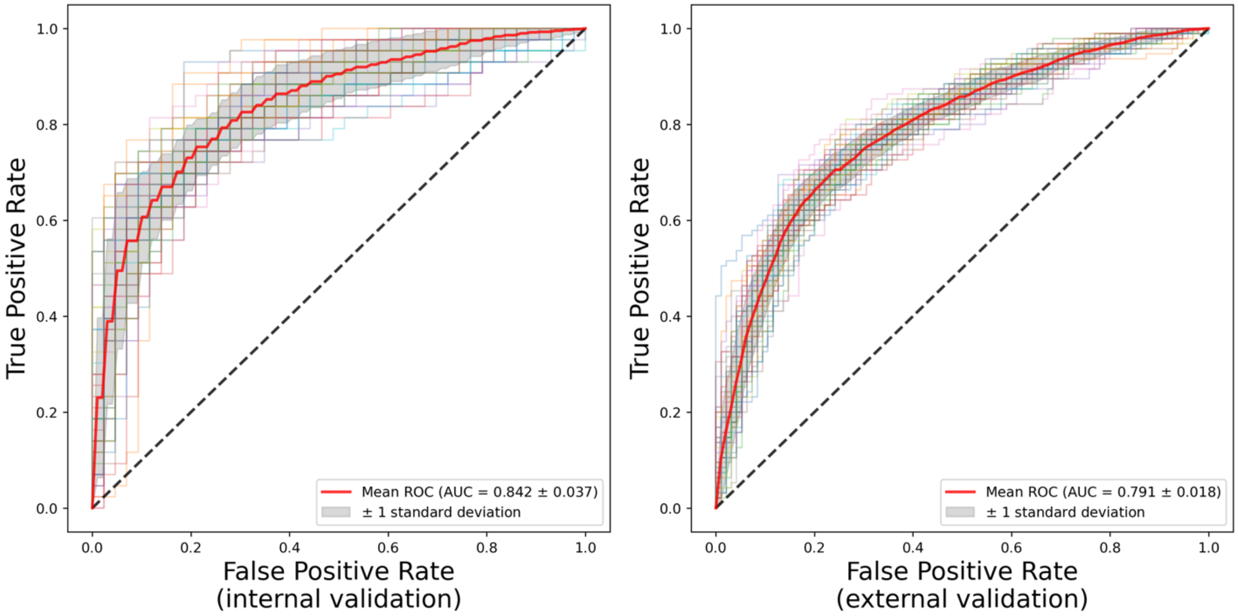

b. ROC curves in internal and external validations using LR.

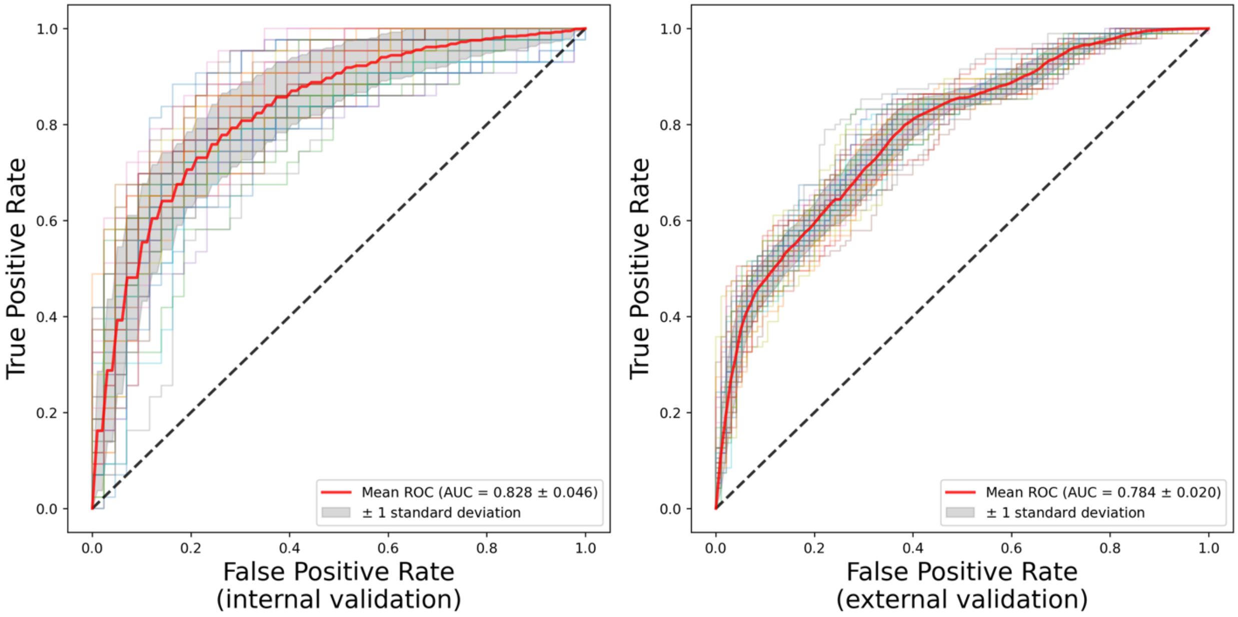

c. ROC curves in internal and external validations using MLP.

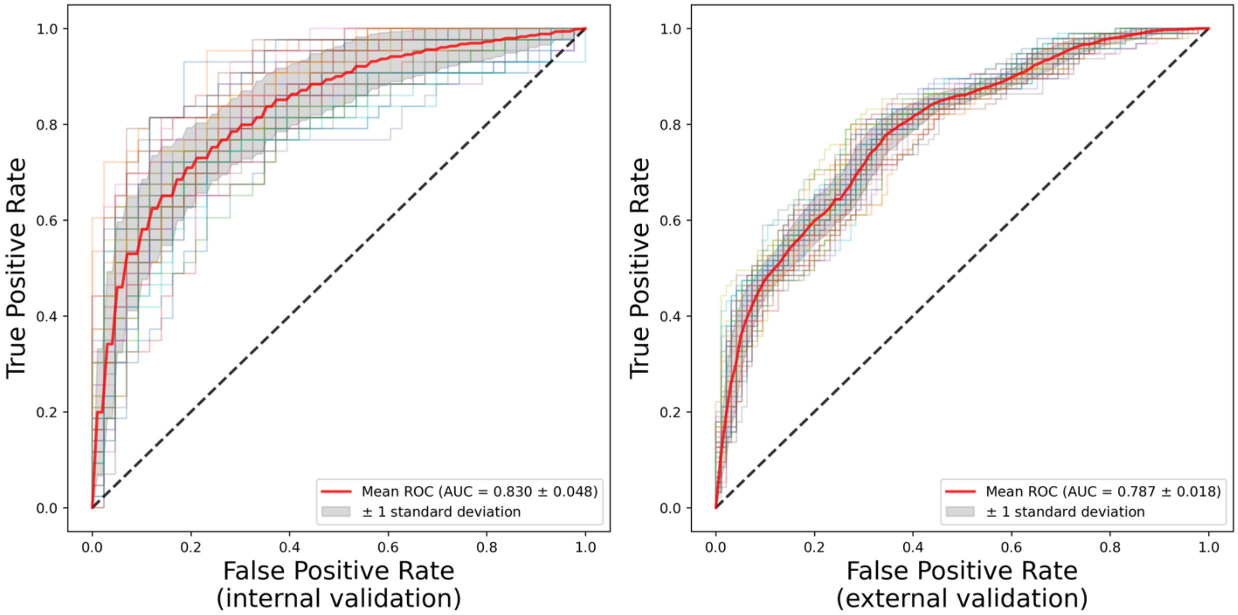

d. ROC curves in internal and external validations using SVM.

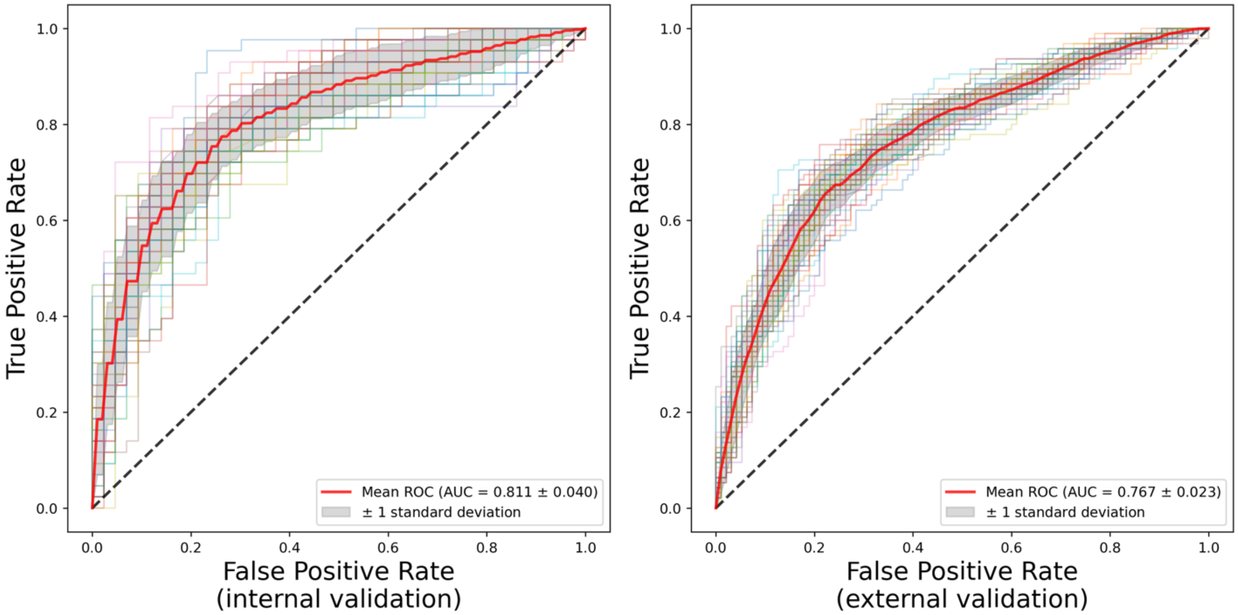

Supplementary Figure 3. Feature importance in recurrent versus non-recurrent classification by MLP, LR, and SVM models.

The box extends from the first quartile to the third quartile of the feature importance values for each feature, with a line at the median. The whiskers extend from the box by 1.5x the interquartile range. Flier points are those past the end of the whiskers.

a. The 20 most important features when experimenting on the original MGB cohort.

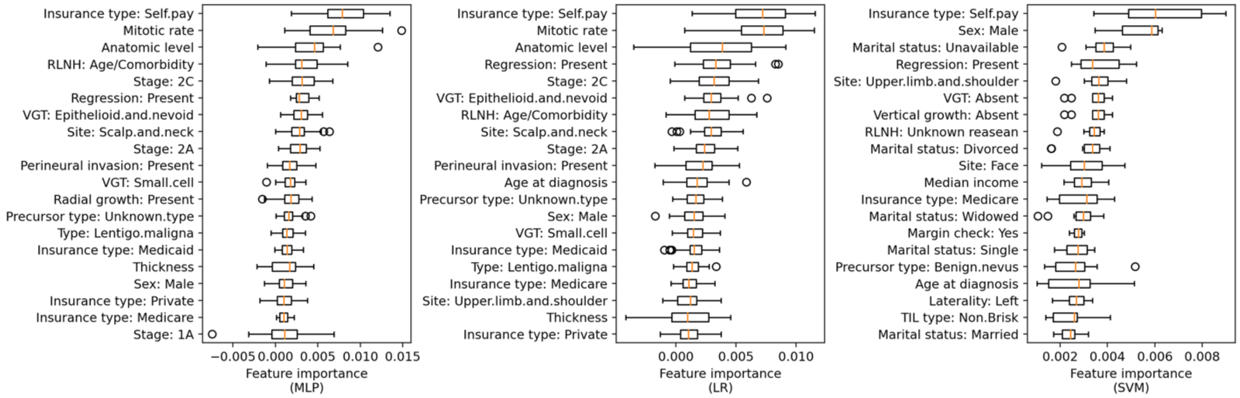

b. The 20 most important features when experimenting on the cohorts only with negative/not indicated regional lymph node histology, known mitotic rate, and known ulceration.

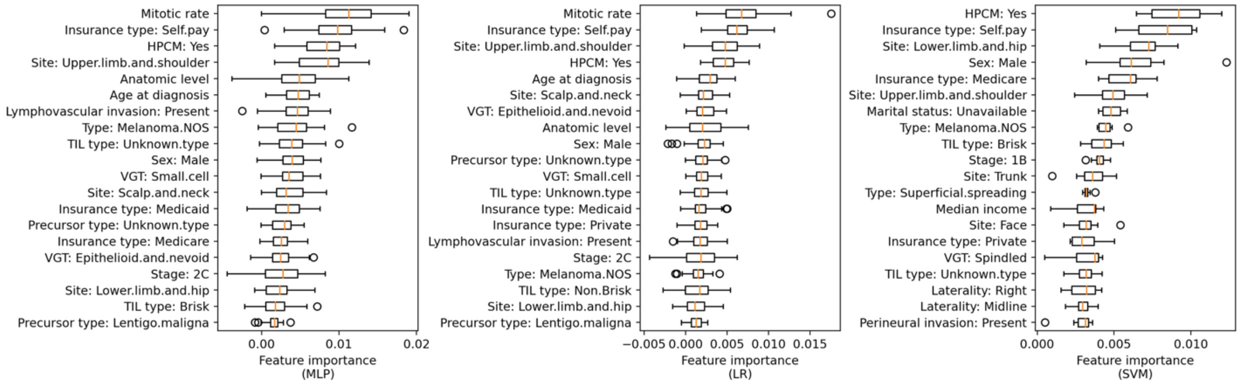

c. The 20 most important features when experimenting on the cohorts only with negative/not indicated regional lymph node histology, and all tumor features available.

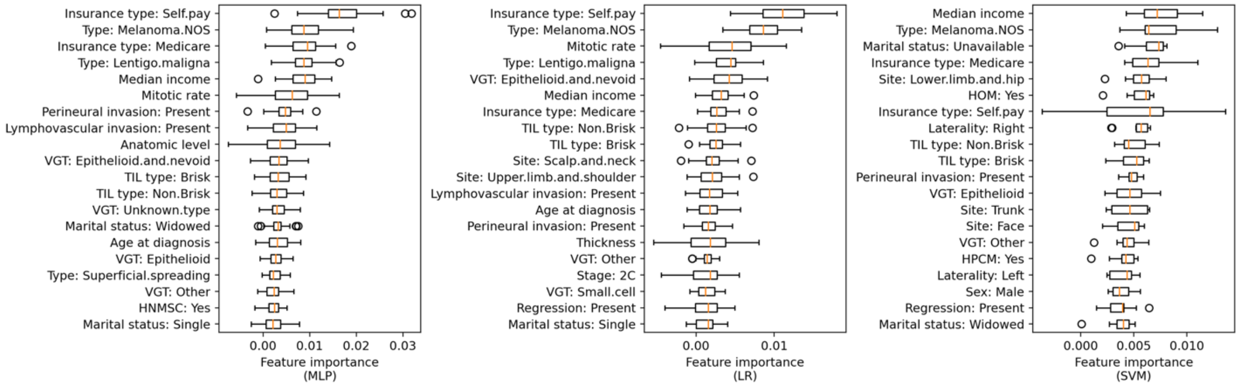

Supplementary Table 7. Recurrence versus non-recurrence classification performance of GB and RF models when removing a subset of Breslow thickness, anatomic level, AJCC stage, and ulceration.

Data		GB		RF	
		AUC	PPV	AUC	PPV
**Original cohorts with all extracted features**	Internal	0.845 CI:0.840-0.850	0.803 CI:0.797-0.810	0.842 CI:0.837-0.847	0.778 CI:0.771-0.785
	External	0.812 CI:0.804-0.819	0.785 CI:0.774-0.795	0.790 CI:0.782-0.797	0.737 CI:0.727-0.748
**Original cohorts without anatomic level, Breslow thickness, and ulceration (AJCC stage was kept)**	Internal	0.830 CI:0.821-0.839	0.788 CI:0.778-0.799	0.831 CI:0.817-0.844	0.776 CI:0.762-0.790
	P-value[a]	<0.001	0.015	0.262	0.844
	External	0.806 CI:0.798-0.814	0.777 CI:0.762-0.792	0.796 CI:0.790-0.803	0.705 CI:0.696-0.715
	P-value[b]	0.342	0.417	0.156	<0.001
**Original cohorts without anatomic level and AJCC stage (Breslow thickness and ulceration were kept)**	Internal	0.842 CI:0.834-0.851	0.793 CI:0.783-0.803	0.842 CI:0.829-0.854	0.781 CI:0.765-0.798
	P-value[a]	0.791	0.105	0.905	0.747
	External	0.809 CI:0.798-0.820	0.774 CI:0.774-0.795	0.784 CI:0.778-0.790	0.732 CI:0.720-0.744
	P-value[b]	0.689	0.253	0.248	0.564

[a] Compared to the internal performance of using all extracted features.

[b] Compared to the external performance of using all extracted features.

Supplementary Figure 4. Feature importance in GB and RF models when removing a subset of Breslow thickness, anatomic level, AJCC stage, and ulceration to examine their collinearity. The box extends from the first quartile to the third quartile of the feature importance values for each feature, with a line at the median. The whiskers extend from the box by 1.5x the interquartile range. Flier points are those past the end of the whiskers.

a. The 20 most important features when experimenting with all extracted features.

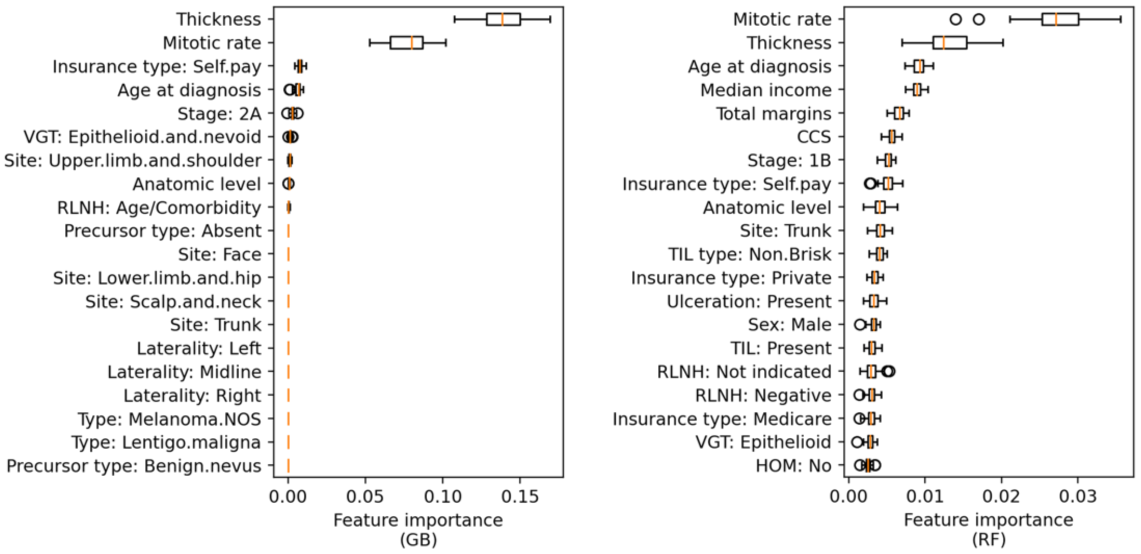

b. The 20 most important features when experimenting without anatomic level, Breslow thickness, and ulceration.

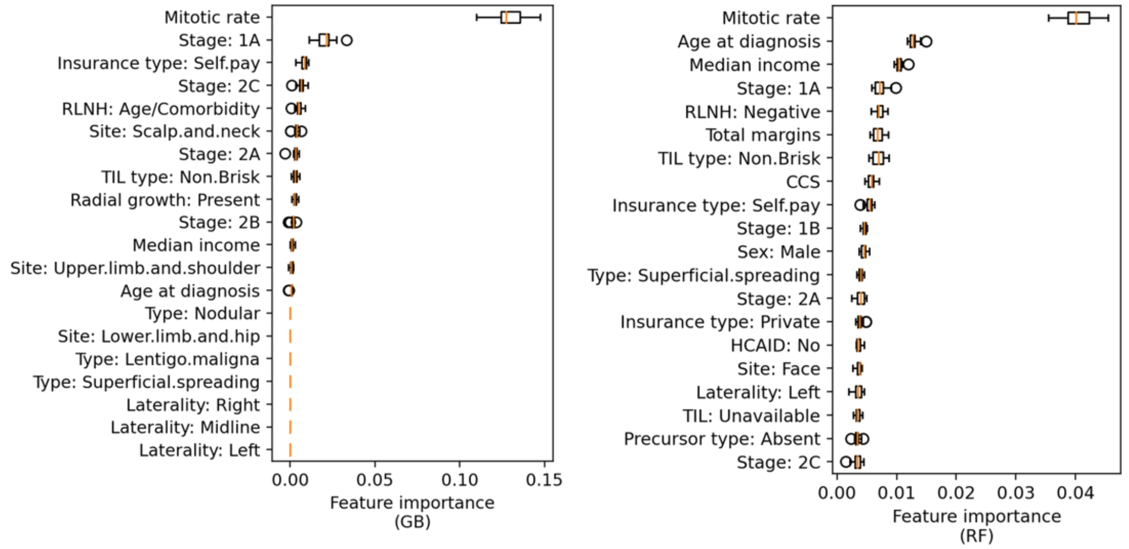

c. The 20 most important features when experimenting without anatomic level and AJCC stage.

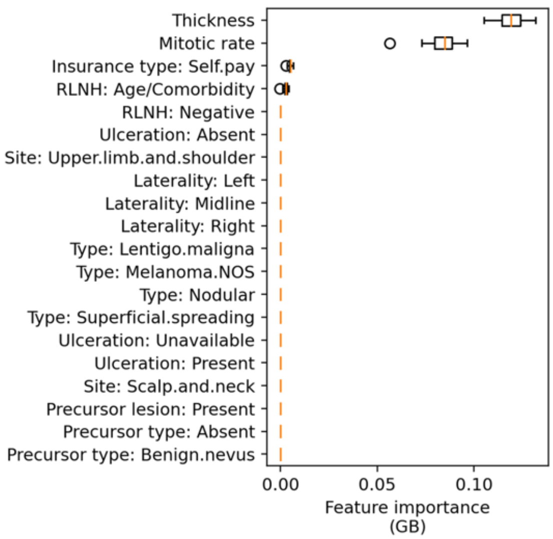

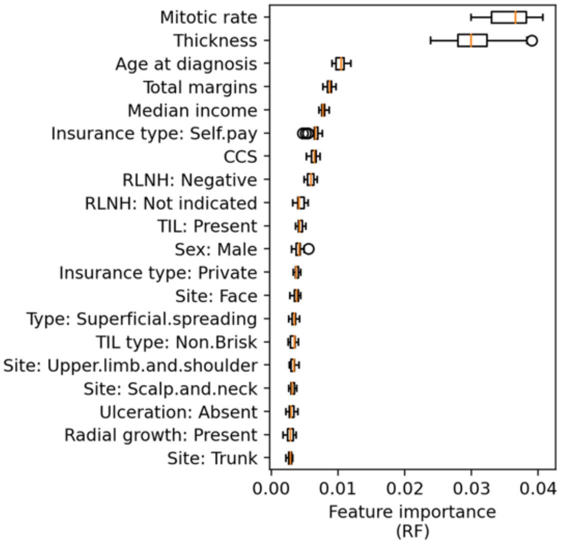

Supplementary Table 8. Time-to-event melanoma recurrence model prediction performances by Coxnet and CoxPH models.

Data	Sample size		Coxnet		CoxPH	
			**Time-dependent AUC**	**Concordance index**	**Time-dependent AUC**	**Concordance index**
**Original cohorts**[a]	**MGB**: 215 recurrences 645 non-recurrences **DFCI**: 95 recurrences 285 non-recurrences	Internal	0.816 CI:0.811-0.822	0.795 CI:0.791-0.799	0.816 CI:0.81-0.821	0.795 CI:0.791-0.799
		External	0.797 CI:0.795-0.798	0.796 CI:0.795-0.797	0.797 CI:0.795-0.798	0.796 CI:0.795-0.797
		P-value[d]	0.002	0.904	0.002	0.904
**Core complete cohorts**[b]	**MGB:** 142 recurrences 516 non-recurrences **DFCI**: 74 recurrences 276 non-recurrences	Internal	0.768 CI:0.757-0.778	0.739 CI:0.732-0.745	0.719 CI:0.714-0.725	0.729 CI:0.722-0.735
		External	0.772 CI:0.77-0.774	0.778 CI:0.776-0.779	0.771 CI:0.768-0.773	0.777 CI:0.776-0.779
		P-value[d]	0.578	<0.001	<0.001	<0.001
**Complete cohorts**[c]	**MGB**: 117 recurrences 370 non-recurrences **DFCI**: 46 recurrences 181 non-recurrences	Internal	0.721 CI:0.714-0.728	0.697 CI:0.691-0.703	0.729 CI:0.721-0.736	0.704 CI:0.698-0.71
		External	0.692 CI:0.689-0.695	0.747 CI:0.745-0.75	0.692 CI:0.688-0.695	0.746 CI:0.743-0.748
		P-value[d]	<0.001	<0.001	<0.001	<0.001

[a] The original cohorts (**Supplementary Table 5**) in which there was no missing values for sex, insurance type, age at diagnosis, HPCM, histological type, tumor site, AJCC stage, Breslow thickness, anatomic level, and laterality (**Supplementary Table 1**).

[b] The "core complete" cohorts were based on the original cohorts with no missing values for core features: negative or not indicated regional lymph node histology, no missing value for ulceration, and no missing value for mitotic rate.

[c] The "complete" cohorts were based on the core complete cohorts with additional constraints: no missing values for median income and all tumor characteristics. Considering the small sample size for time-to-vent prediction, 2-fold cross-validation was used for internal validation.

[d] P-value: comparison between the internal validation and the external validation.

[e] CI: 95% confidence interval.

Supplementary Figure 5. Feature importance in time-to-event recurrence prediction by Coxnet and CoxPH models.

The box extends from the first quartile to the third quartile of the feature importance values for each feature, with a line at the median. The whiskers extend from the box by 1.5x the interquartile range. Flier points are those past the end of the whiskers.

a. The 20 most important features when experimenting on the original cohorts.

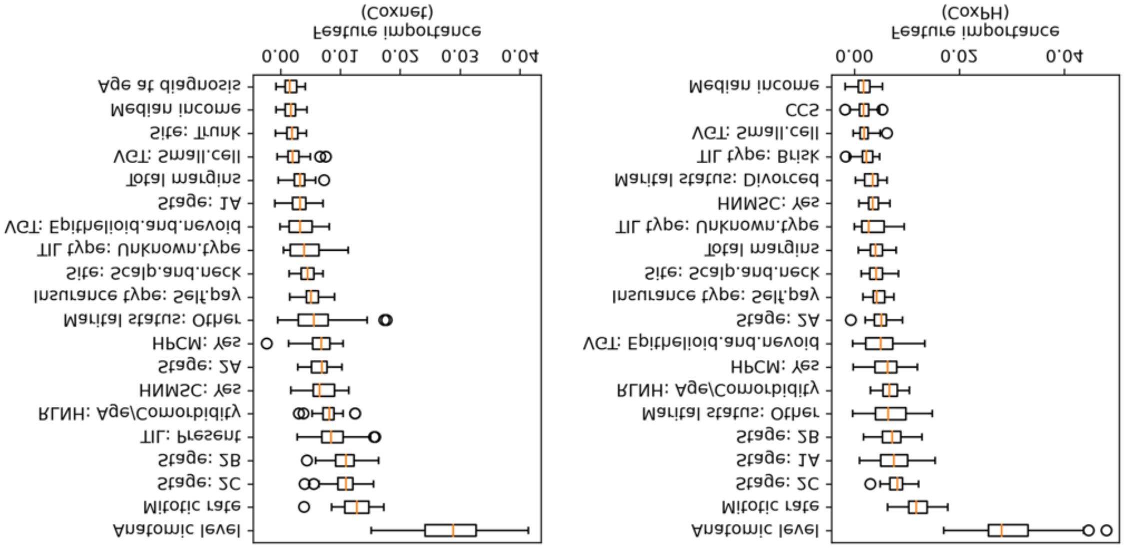

b. The 20 most important features when experimenting on the core complete cohorts: negative or not indicated regional lymph node histology, known mitotic rate, and known ulceration.

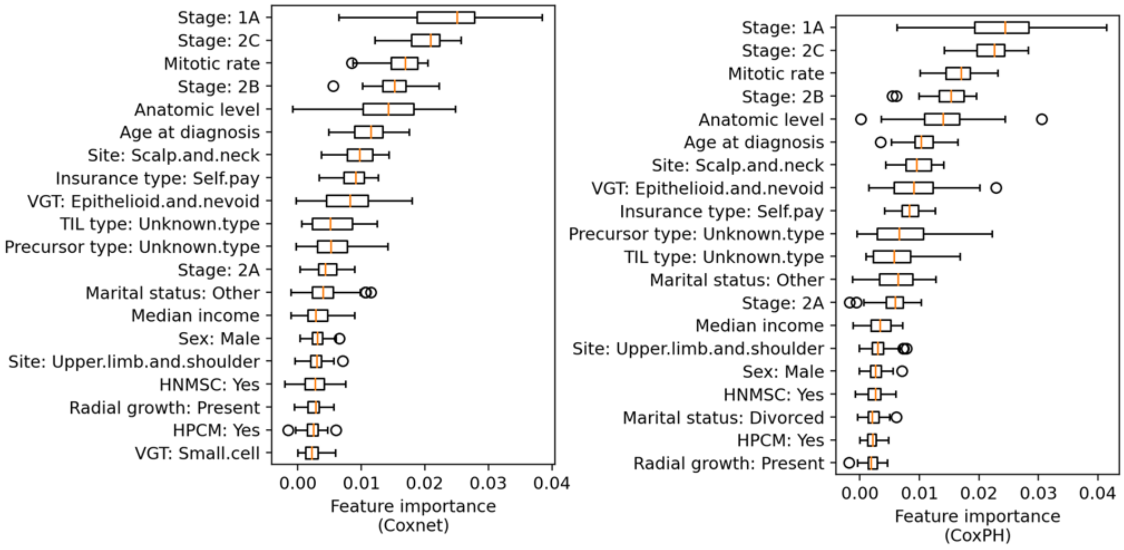

c. The 20 most important features when experimenting on the complete cohorts: negative not indicated regional lymph node histology, known median income, tumor characteristics known.

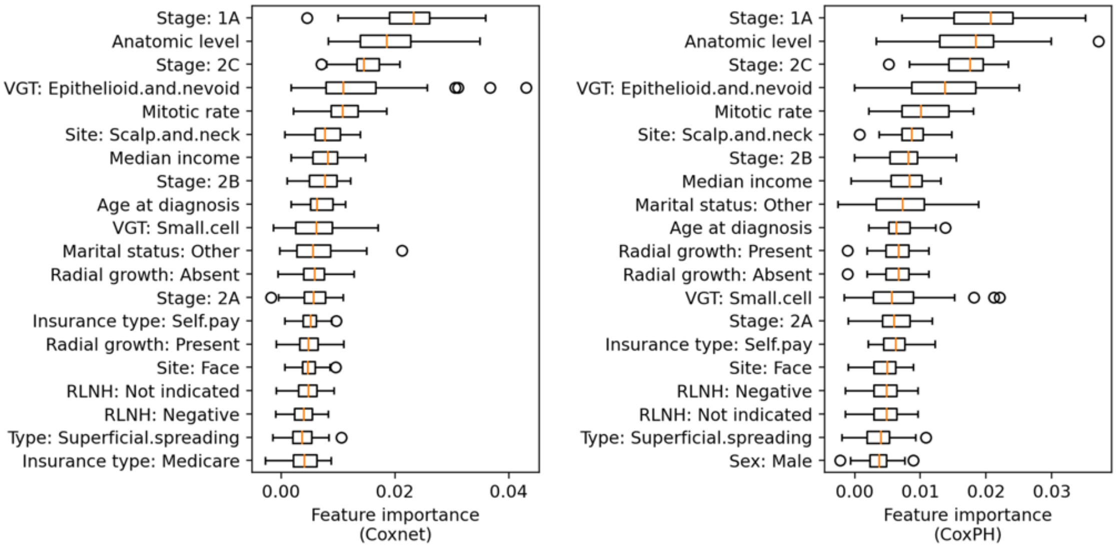

Supplementary Table 9. Recurrence prediction using GB-T and RF-T models when removing a subset of Breslow thickness, anatomic level, AJCC stage, and ulceration.

**Data**		**GB-T**		**RF-T**	
		**Time-dependent AUC**	**Concordance index**	**Time-dependent AUC**	**Concordance index**
**Original cohorts with all extracted features**	Internal	0.853 CI:0.849-0.857	0.820 CI:0.816-0.824	0.845 CI:0.841-0.850	0.813 CI:0.809-0.816
	External	0.820 CI:0.819-0.821	0.809 CI:0.808-0.810	0.810 CI:0.809-0.811	0.807 CI:0.807-0.808
**Original cohorts without Breslow thickness, anatomic level, and ulceration (AJCC stage was kept)**	Internal	0.856 CI:0.850-0.862	0.818 CI:0.811-0.825	0.843 CI:0.836-0.850	0.811 CI:0.805-0.817
	P-value[a]	0.516	0.690	0.482	0.532
	External	0.809 CI:0.807-0.811	0.809 CI:0.807-0.811	0.807 CI:0.805-0.809	0.805 CI:0.803-0.806
	P-value[b]	<0.001	0.431	0.037	0.024
**Original cohorts without anatomic level and AJCC stage (Breslow thickness and ulceration were kept)**	Internal	0.854 CI:0.848-0.860	0.821 CI:0.814-0.828	0.846 CI:0.839-0.853	0.814 CI:0.809-0.820
	P-value[a]	0.579	0.766	0.913	0.517
	External	0.818 CI:0.817-0.820	0.808 CI:0.806-0.809	0.809 CI:0.807-0.811	0.805 CI:0.804-0.807
	P-value[b]	0.253	0.895	0.570	0.032

[a] Compared to the internal performance of using all extracted features.

[b] Compared to the external performance of using all extracted features.

Supplementary Figure 6. Prediction performance of GB-T and RF-T models when removing a subset of anatomic level, Breslow thickness, AJCC stage, and ulceration.
The box extends from the first quartile to the third quartile of the feature importance values for each feature, with a line at the median. The whiskers extend from the box by 1.5x the interquartile range. Flier points are those past the end of the whiskers.

a. The 20 most important features when experimenting with all extracted features.

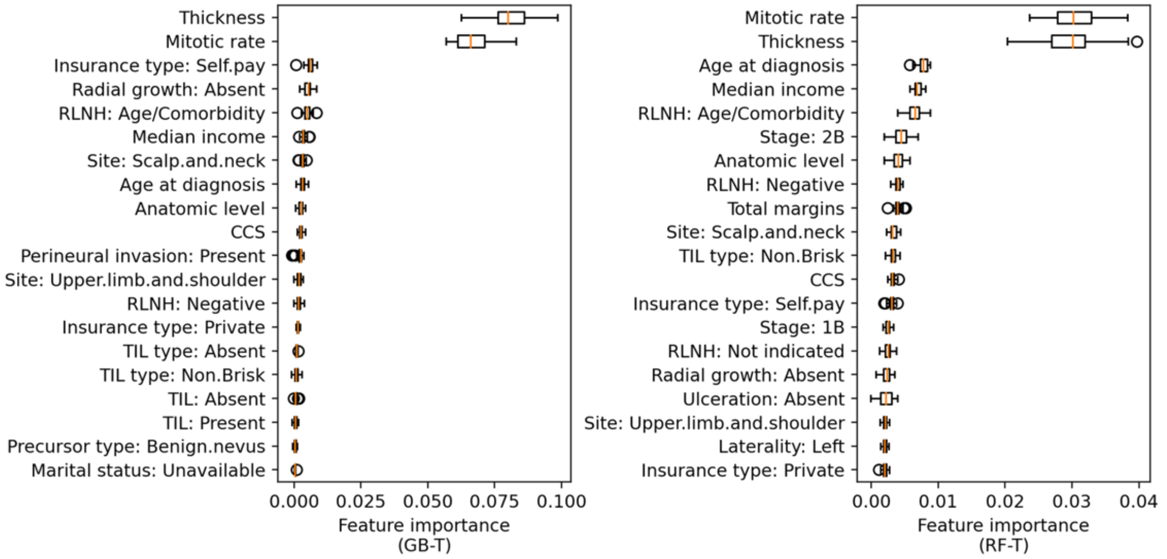

b. The 20 most important features when experimenting without anatomic level, Breslow thickness, and ulceration.

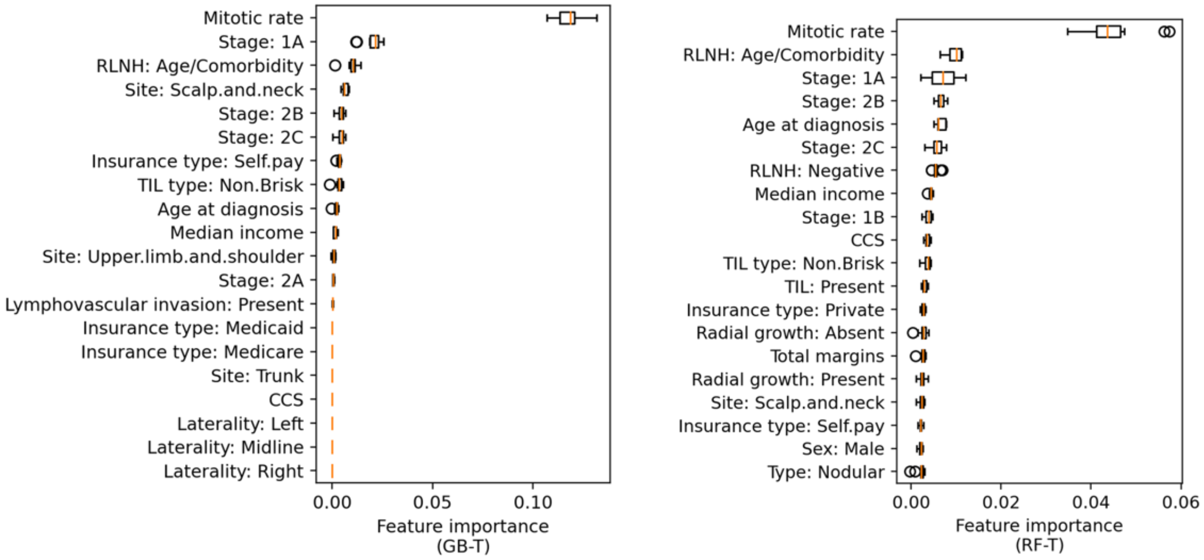

c. The 20 most important features when experimenting without anatomic level and AJCC stage.

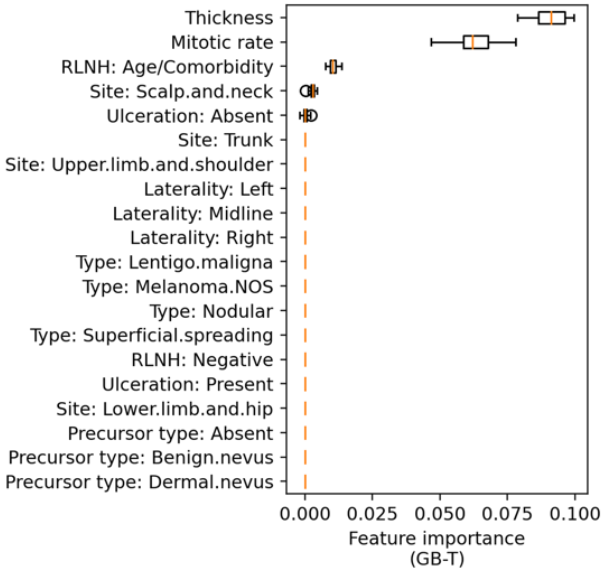

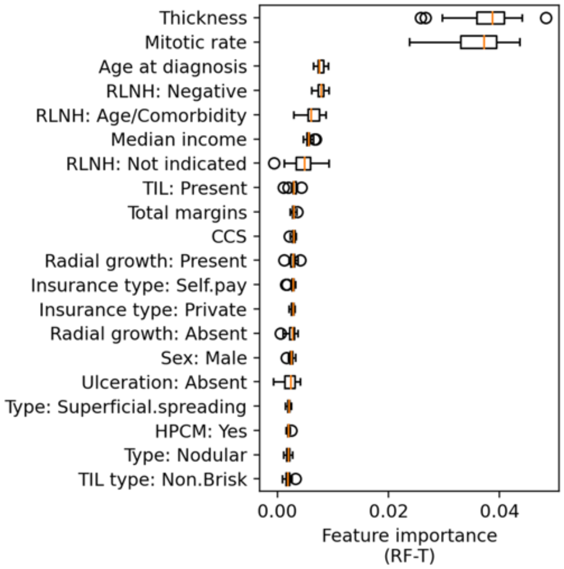

Supplementary Table 10. ICD codes used to identify cutaneous autoimmune diseases.

Index	disease	ICD-10	ICD-9
1	Dermatomyositis	M33 (Dermatopolymyositis)	710.3 (Dermatomyositis)
2	Psoriasis	L40 (psoriasis)	696.0 (arthropathic psoriasis)<br>696.1 (Other psoriasis)
3	Lichen planus	L43 (Lichen planus)	697.0 (Lichen planus)
4	Vitiligo	L80 (Vitiligo)	709.01 (Vitiligo)
5	Bullous pemphigoid	L12 (Pemphigoid)	694.5 (Pemphigoid)
6	Pemphigus	L10 (Pemphigus)	694.4 (Pemphigus)
7	Atopic dermatitis	L20 (Atopic dermatitis)	691.8 (Other atopic dermatitis and related conditions)
8	Seborrheic dermatitis	L21 (Seborrheic dermatitis)	690.1 (Seborrheic dermatitis)
9	Lupus erythematosus	L93 (Lupus erythematosus)	695.4 (Lupus erythematosus)
10	Alopecia areata	L63 (Alopecia areata)	704.09 (Other alopecia)
11	Morphea	L90.0 (Lichen sclerosus et atrophicus)<br>L94.0 (Localized scleroderma)<br>L94.3 (Sclerodactyly)	701.0 (Circumscribed scleroderma)
12	Dermatitis herpetiformis	L13.0 (Dermatitis herpetiformis)	694.0 (Dermatitis herpetiformis)
13	Vasculitis	L95.8 (Other vasculitis limited to the skin)<br>L95.9 (Vasculitis limited to the skin, unspecified)	709.1 (Vascular disorders of skin)
14	Mucositis	K12 (Stomatitis and related lesions)	528.00 (Stomatitis and mucositis, unspecified)<br>528.09 (Other stomatitis and mucositis)<br>528.2 (Oral aphthae)
15	Pyoderma gangrenosum	L88 (Pyoderma gangrenosum)	686.01 (Pyoderma gangrenosum)
16	Hidradenitis suppurativa	L73.2 (Hidradenitis suppurativa)	705.83 (Hidradenitis)
17	Erythema nodosum	L52 (Erythema nodosum)	695.2 (Erythema nodosum)
18	Linear IgA	L13.8 (Other specified bullous disorders)	694.8 (Other specified bullous dermatoses)

Supplementary Table 11. ICD codes used to identify systemic autoimmune diseases.

Index	Disease	ICD-10	ICD-9
1	Rheumatoid arthritis	M05 (Rheumatoid arthritis with rheumatoid factor)<br>M06 (Other rheumatoid arthritis)<br>M08 (Juvenile rheumatoid arthritis)	714.0 (Rheumatoid arthritis)
2	Systemic sclerosis	M34 (Systemic sclerosis)	710.1 (Systemic sclerosis)
3	Systemic Lupus	M32 (Systemic lupus erythematosus)	710.0 (Systemic lupus erythematosus)
4	Inflammatory bowel disease	K50 (Crohn's disease)<br>K51 (Ulcerative colitis)<br>K52 (Other and unspecified noninfective gastroenteritis and colitis)	555.9 (Crohn's disease of unspecified site)<br>556.6 (Universal ulcerative chronic colitis)<br>556.9 (Ulcerative colitis, unspecified)<br>558.9 (Other and unspecified noninfectious gastroenteritis and colitis)
5	Ankylosing spondylitis	M45 (Ankylosing Spondylitis)	720.0 (Ankylosing spondylitis)
6	Sicca syndrome	M35.0 (Sjögren syndrome)	710.2 (Sicca syndrome)
7	Polymyalgia rheumatica	M35.3 (Polymyalgia rheumatica)	725 (Polymyalgia rheumatica)
8	Mixed connective tissue	M35.9 (Systemic involvement of connective tissue, unspecified)	710.9 (Unspecified diffuse connective tissue disease)
9	Type 1 diabetes	E10 (Type 1 diabetes mellitus)	250.x1 (Diabetes mellitus, type I, not stated as uncontrolled)<br>250.x3 (Diabetes mellitus, type I, uncontrolled) x = 0 to 9.
10	Myasthenia gravis	G70.0 (Myasthenia gravis)	358.00 (Myasthenia gravis without acute exacerbation)<br>358.01 (Myasthenia gravis with acute exacerbation)
11	Graves' disease	E05.00 (Thyrotoxicosis with diffuse goiter without thyrotoxic crisis or storm)<br>E05.01 (Thyrotoxicosis with diffuse goiter with thyrotoxic crisis or storm)	242.00 (Toxic diffuse goiter with no crisis or storm)<br>242.01 (Toxic diffuse goiter crisis or storm)
12	Autoimmune thyroiditis	E06.3 (Autoimmune thyroiditis)	245.2 (Chronic lymphocytic thyroiditis)
13	Addison’s disease	E27.1 (Addison’s disease)<br>E27.2 (Addisonian crisis)<br>E27.3 (Drug-induced adrenocortical insufficiency)<br>E27.4 (Other and unspecified adrenocortical insufficiency)	255.41 (Glucocorticoid deficiency)
14	Autoimmune Hepatitis	K75.4 (Autoimmune hepatitis)	571.42 (Autoimmune hepatitis)
15	Celiac disease	K90.0 (Celiac disease)	579.0 (Celiac disease)
16	Vasculitis	I77.6 (Arteritis, unspecified)<br>M30.1 (Polyarteritis with lung involvement)<br>M31 (Other necrotizing vasculopathies)	446.0 (Polyarteritis nodosa)<br>446.20 (Hypersensitivity angiitis, unspecified)<br>446.21 (Goodpasture's syndrome)

			446.29 (Other specified hypersensitivity angiitis)<br>446.3 (Lethal midline granuloma)<br>446.4 (Wegener's granulomatosis)<br>446.5 (Giant cell arteritis)<br>446.7 (Takayasu's disease)<br>447.5 (Necrosis of artery)<br>447.6 (Arteritis, unspecified)
17	Sarcoidosis	D86 (Sarcoidosis)	135 (Sarcoidosis)
18	Autoimmune hemolytic anemia	D59.0 (Drug-induced autoimmune hemolytic anemia)<br>D59.1 (Other autoimmune hemolytic anemias)	283.0 (Autoimmune hemolytic anemias)
19	Biliary cirrhosis	K74.3 (Primary biliary cirrhosis)<br>K74.4 (Secondary biliary cirrhosis)<br>K74.5 (Biliary cirrhosis, unspecified)	571.6 (Biliary cirrhosis)
20	Guillain-barre syndrome	G61.0 (Guillain-barre syndrome)	357.0 (Guillain-barre syndrome)
21	Reiter's disease	M02.3 (Reiter's disease)	099.3 (Reiter's disease)
22	Hypothyroidism	E03.8 (Other specified hypothyroidism)<br>E03.9 (Hypothyroidism, unspecified)	244.8 (Other specified acquired hypothyroidism)<br>244.9 (Hypothyroidism, unspecified)
23	Multiple Sclerosis	G35 (Multiple Sclerosis)	340 (Multiple Sclerosis)

Supplementary Table 12. ICD codes used to identify medical history features.

**Disease**	**ICD-10**	**ICD-9**
Other non-melanoma skin cancer	C44 (Other and unspecified malignant neoplasm of skin)<br>C46.0 (Kaposi's sarcoma of skin)<br>C4A (Merkel cell carcinoma)<br>Z85.828 (Personal history of other malignant neoplasm of skin)	173 (Other and unspecified malignant neoplasm of skin)<br>V10.83 (Personal history of other malignant neoplasm of skin)
Situ or benign neoplasm of skin	D04 (Carcinoma in situ of skin)<br>D18.01 (Hemangioma of skin and subcutaneous tissue)<br>D23.5 (Other benign neoplasm of skin of trunk)<br>D48.5 (Neoplasm of uncertain behavior of skin)	214.0 (Lipoma of skin and subcutaneous tissue of face)<br>214.1 (Lipoma of other skin and subcutaneous tissue)<br>216 (Benign neoplasm of skin)<br>232 (Carcinoma in situ of skin)<br>228.01 (Hemangioma of skin and subcutaneous tissue)<br>238.2 (Neoplasm of uncertain behavior of skin)<br>239.2 (Neoplasm of unspecified behavior of bone, soft tissue, and skin)
