# Supplementary material for: Prediction of early-stage melanoma recurrence using clinical and histopathologic features
Source: NPJ Precis Oncol. 2022 Oct 31;6:79. doi: 10.1038/s41698-022-00321-4 (PMC9622809; doi:10.1038/s41698-022-00321-4)
Supplement: Supplementary file 2 — REPORTING SUMMARY [file 41698_2022_321_MOESM2_ESM.pdf]

nature portfolio

Corresponding author(s): Yevgeniy R. Semenov

Last updated by author(s): Oct 2, 2022

Reporting Summary

Nature Portfolio wishes to improve the reproducibility of the work that we publish. This form provides structure for consistency and transparency in reporting. For further information on Nature Portfolio policies, see our Editorial Policies and the Editorial Policy Checklist.

Statistics

For all statistical analyses, confirm that the following items are present in the figure legend, table legend, main text, or Methods section.

n/a	Confirmed	
☐	☒	The exact sample size (*n*) for each experimental group/condition, given as a discrete number and unit of measurement
☐	☒	A statement on whether measurements were taken from distinct samples or whether the same sample was measured repeatedly
☐	☒	The statistical test(s) used AND whether they are one- or two-sided<br>*Only common tests should be described solely by name; describe more complex techniques in the Methods section.*
☐	☒	A description of all covariates tested
☐	☒	A description of any assumptions or corrections, such as tests of normality and adjustment for multiple comparisons
☐	☒	A full description of the statistical parameters including central tendency (e.g. means) or other basic estimates (e.g. regression coefficient) AND variation (e.g. standard deviation) or associated estimates of uncertainty (e.g. confidence intervals)
☐	☒	For null hypothesis testing, the test statistic (e.g. *F*, *t*, *r*) with confidence intervals, effect sizes, degrees of freedom and *P* value noted<br>*Give P values as exact values whenever suitable.*
☒	☐	For Bayesian analysis, information on the choice of priors and Markov chain Monte Carlo settings
☒	☐	For hierarchical and complex designs, identification of the appropriate level for tests and full reporting of outcomes
☒	☐	Estimates of effect sizes (e.g. Cohen's *d*, Pearson's *r*), indicating how they were calculated

Our web collection on statistics for biologists contains articles on many of the points above.

Software and code

Policy information about availability of computer code

Data collection	R version 4.2.1; python 3.8.12
Data analysis	python 3.8.12; scikit-learn 0.24.1; numpy 1.20.2; scikit-survival 0.17.2

For manuscripts utilizing custom algorithms or software that are central to the research but not yet described in published literature, software must be made available to editors and reviewers. We strongly encourage code deposition in a community repository (e.g. GitHub). See the Nature Portfolio guidelines for submitting code & software for further information.

Data

Policy information about availability of data

All manuscripts must include a data availability statement. This statement should provide the following information, where applicable:

- Accession codes, unique identifiers, or web links for publicly available datasets
- A description of any restrictions on data availability
- For clinical datasets or third party data, please ensure that the statement adheres to our policy

All relevant data are available from the corresponding author. The summary data supporting the findings of this study are available within the article and/or its supplementary materials. The patient data generated for this study can only be shared per specific institutional review board (IRB) requirements. Upon a reasonable request, a data-sharing agreement can be initiated following institution-specific guidelines.
The software code is available at https://github.com/SemenovLab/early-stage-melanoma-recurrence-prediction.

Human research participants

Policy information about studies involving human research participants and Sex and Gender in Research.

Reporting on sex and gender	Males and females diagnosed with Stage I or Stage II melanoma at Mass General Brigham (MGB) and Dana Farber Cancer Institute (DFCI) between January 2000 and February 2020 were included in this study. The sex was extracted from electronic health records at the two institutions, and 56% of the study population were male. Detailed characteristics are presented in Table 1 in the manuscript.
Population characteristics	We identified 1,720 stage I/II cutaneous melanomas, with 1,172 (68%) melanomas from MGB and 548 (32%) melanomas from DFCI. The median follow-up for the entire cohort was 7.2 (IQR: 3.6-11.6) years. Overall, 310 out of 1,720 (18%) melanomas recurred, among which 151 (48.7%) were distant recurrences. The median time from diagnosis to recurrence was 1.9 (IQR: 0.9-3.9) years. There was a small difference in the distribution of the year of diagnosis between the recurrent group and non-recurrent group (p-value: 0.02). However, the mean and median year of diagnosis were identical for both groups (2010). The recurrent group had a higher mortality rate (49% vs 22%, p-value <0.001) and was older at the time of diagnosis (65 vs 60 years old, p-value <0.001) when compared to the non-recurrent group. The percentage of males in the recurrent group was higher (64% vs 55%, p-value: 0.004) than in the non-recurrent group. Most of the population self-identified as Caucasian in both groups (recurrence: 98%; non-recurrence: 99%). Among the 310 recurrent melanomas, the respective number of cases recurred within five years and seven years after the diagnosis of the primary melanoma is 255 (82%) and 285 (92%).
Recruitment	Stage I/II cutaneous melanomas with no evidence of metastasis at the time of diagnosis and of the following histological types were included: lentigo maligna, nodular, superficial spreading, and melanoma NOS (not otherwise specified). Acral, mucosal, and uveal melanomas were excluded from the study. Melanoma histology (confirmed by ICD-O-3 codes), diagnosis date of the primary melanoma, and recurrence date were extracted from the cancer registrars at both institutions.
Ethics oversight	The Mass General Brigham Institutional Review Board (IRB) approved the study (Protocol # 2020P002179). The need for consent was waived by the IRB as the study meets the criteria for exemption 45 CFR 46.104(d)(#).

Note that full information on the approval of the study protocol must also be provided in the manuscript.

Field-specific reporting

Please select the one below that is the best fit for your research. If you are not sure, read the appropriate sections before making your selection.

☒ Life sciences ☐ Behavioural & social sciences ☐ Ecological, evolutionary & environmental sciences

For a reference copy of the document with all sections, see nature.com/documents/nr-reporting-summary-flat.pdf

Life sciences study design

All studies must disclose on these points even when the disclosure is negative.

Sample size	Patients diagnosed with Stage I or Stage II melanoma at Mass General Brigham (MGB) and Dana Farber Cancer Institute (DFCI) between January 2000 and February 2020 were included in this study. We identified 1,720 stage I/II cutaneous melanomas, with 1,172 (68%) melanomas from MGB and 548 (32%) melanomas from DFCI. Sample size was calculated and described in the manuscript.
Data exclusions	Acral, mucosal, and uveal melanomas were excluded from the study. Melanomas without pathology reports available were excluded. Cutaneous stage I/II melanomas with the following histological types were included: lentigo maligna, nodular, superficial spreading, and melanoma NOS (not otherwise specified).
Replication	To assess the machine learning algorithms in early-stage melanoma recurrence prediction, all models were evaluated internally and externally: 1) 5-fold cross-validation of the MGB cohort; 2) the MGB cohort for training and the DFCI cohort for testing independently.
Randomization	The non-recurrent melanomas were categorized into two groups: one group with a minimum of 5-year follow-up duration (to minimize the risk of false non-recurrences); another group 3:1 matched to the recurrent melanomas in terms of follow-up duration. The first group combined with the recurrent melanomas was designed for the binary recurrence classification tasks. The second group combined with the recurrent melanomas was used for the time-to-event recurrence prediction tasks.
Blinding	Inclusion and exclusion criteria were used to collect the study population. Manual chart review was conducted to extract features of interests from electronic health records. A detailed data collection design was described in the manuscript.

Reporting for specific materials, systems and methods

We require information from authors about some types of materials, experimental systems and methods used in many studies. Here, indicate whether each material, system or method listed is relevant to your study. If you are not sure if a list item applies to your research, read the appropriate section before selecting a response.

Materials & experimental systems

n/a	Involved in the study
☒	☐ Antibodies
☒	☐ Eukaryotic cell lines
☒	☐ Palaeontology and archaeology
☒	☐ Animals and other organisms
☐	☒ Clinical data
☒	☐ Dual use research of concern

Methods

n/a	Involved in the study
☒	☐ ChIP-seq
☒	☐ Flow cytometry
☒	☐ MRI-based neuroimaging

Clinical data

Policy information about clinical studies

All manuscripts should comply with the ICMJE guidelines for publication of clinical research and a completed CONSORT checklist must be included with all submissions.

Clinical trial registration: This study was not a clinical trial.

Study protocol: Protocol # 2020P002179

Data collection: Patients diagnosed with Stage I or Stage II melanoma at Mass General Brigham and Dana Farber Cancer Institute between January 2000 and February 2020 were included in this study. We extracted 36 demographic, clinical, and histopathologic features from electronic health records.

Outcomes: Each included melanoma was labeled as having a recurrence or not, and its recurrence date or last follow-up date was recorded. The primary outcomes are the recurrence versus non-recurrence classification and the time-to-event recurrence prediction using machine learning. The secondary analysis is to investigate the importance ranking of all extracted features.
